# Exploring the Role of TRIP-Brs in Human Breast Cancer: An Investigation of Expression, Clinicopathological Significance, and Prognosis

**DOI:** 10.1016/j.omto.2020.09.003

**Published:** 2020-09-16

**Authors:** Raj Kumar Mongre, Chandra Bhushan Mishra, Samil Jung, Beom Suk Lee, Nguyen Thi Ngoc Quynh, Nguyen Hai Anh, Davaajragal Myagmarjav, Taeyeon Jo, Myeong-Sok Lee

**Affiliations:** 1 Molecular Cancer Biology Laboratory, Cellular Heterogeneity Research Center, Department of Biosystem, Sookmyung Women’s University, Hyochangwon gil-52, Yongsan-Gu, Seoul 140-742, Republic of Korea; 2 College of Pharmacy, Sookmyung Women’s University, Hyochangwon gil-52, Yongsan-Gu, Seoul 140-742, Republic of Korea

**Keywords:** transcription factors, TRIP-Brs, BRCA, mutation, clinicopathological, patient survival, tumor-infiltrating immune cells, KEGG pathways

## Abstract

TRIP-Brs, a group of transcription factors (TFs) that modulate several mechanisms in higher organisms. However, the novel paradigm to target TRIP-Brs in specific cancer remains to be deciphered. In particular, comprehensive analysis of TRIP-Brs in clinicopathological and patients’ prognosis, especially in breast cancer (BRCA), is being greatly ignored. Therefore, we explored the key roles of TRIP-Br expression, modulatory effects, mutations, immune infiltration, and prognosis in BRCA using multidimensional approaches. We found elevated levels of TRIP-Brs in numerous cancer tissues than normal. Higher expression of TRIP-Br-2/4/5 was shown to be positively associated with lower survival, tumor grade, and malignancy of patients with BRCA. Additionally, higher TRIP-Br-3/4 were also significantly linked with worse/short survival of BRCA patients. TRIP-Br-1/4/5 were significantly overexpressed and enhanced tumorigenesis in large-scale BRCA datasets. The mRNA levels of TRIP-Brs have been also correlated with tumor immune infiltrate in BRCA patients. In addition, TRIP-Brs synergistically play a pivotal role in central carbon metabolism, cancer-associated pathways, cell cycle, and thyroid hormone signaling, which evoke that TRIP-Brs may be a potential target for the therapy of BRCA. Thus, this investigation may lay a foundation for further research on TRIP-Br-mediated management of BRCA.

## Graphical Abstract

**Figure:**
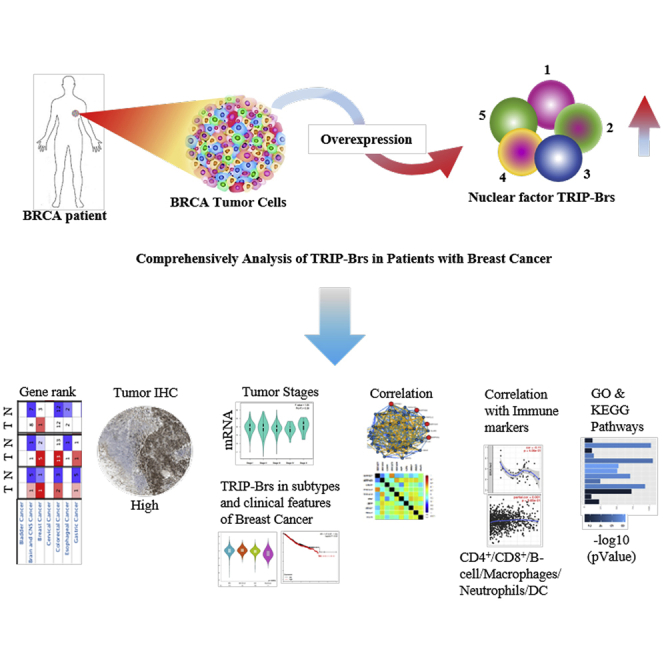

## Teaser

Higher expression of TRIP-Brs was observed in breast cancer patients via modulating the multiple signaling pathways associated with the fate of cancer cells. Higher expression of TRIP-Brs was positively associated with lower survival, tumor grade, and malignancy of BRCA patients. TRIP-Brs appeared as valuable biomarkers for prognosis of breast cancer.

## Introduction

Breast cancer (BRCA) is referred as the most deadly disease, and it causes approximately 6% deaths among all cancer-associated deaths worldwide.^1^ Nowadays, several therapeutic approaches are being used to cure BRCA, such as surgery, chemotherapy, radiation therapy, etc.^2^ However, these therapeutic strategies are suffering from the high risk of long-term side effects. Therefore, it is a dire need to explore an effective therapeutic target for successful management of BRCA. To better understand, BRCA can be divided into four subtypes according to levels of epidermal growth factor receptor (EGFR), progesterone receptor (PR), cytokeratins (CKs), estrogen receptor (ER), and human EGFR-2 (HER2): (1) luminal-A: ER^positive^/PR^positive^/HER2^negative^; (2) luminal-B: ER^positive^/PR^positive^/HER2^positive^; (3) basal-like subtypes: ER^negative^/PR^negative^/HER2^negative^/CK-5/6^positive^/EGFR^positive^; (4) HER2-overexpressing subtype: ER^negative^/PR^negative^/HER2^positive^.^3^ In BRCA management, ER, PR, and HER2 have been opted as a classical prognostic biomarker that played a key function in the diagnosis and therapy of BRCA.^3^^,^^4^ Despite great development in BRCA detection, treatment, and management, it has been found that around 5% to 10% of patients exhibit metastatic tumors after the first treatment, and about ⅕ of all survive 5 years.^1^ Tumor heterogeneity is a common hallmark of most of cancers, including breast carcinoma.^5^ Due to tumor heterogeneity, several biomarkers are suffering from limitations associated with patients’ survival and prognosis. Therefore, there is an urgent need to explore potential biomarkers as survival and prognostic indicators for effective management of BRCA.

Currently, five TRIP-Br nuclear transcription factors have been invented as gene series: TRIP-Br-1, TRIP-Br-2, TRIP-Br-3, TRIP-Br-4 and TRIP-Br-5.^6^, ^7^, ^8^ The nuclear factor TRIP-Brs have multiple distinct functions in BRCA progression.^9^, ^10^, ^11^, ^12^, ^13^ TRIP-Br-1 is known to be found with an overexpression during BRCA initiation.^11^ It is reported that TRIP-Br-1 acts as a master regulator of cell growth that interacts with several proteins, such as E2F1, DP-1, I-mfa, Fbxw7, CDK4, p16^INKAa^, AC1, XIAP, and RING-domain E3 ubiquitin ligase.^10^, ^11^, ^12^, ^13^ It has also been demonstrated that the N terminus of TRIP-Br-1 binds with BIR domain of XIAP,^11^ and TRIP-Br-1/XIAP both act as key proteins in BRCA cells under the influence of nutrient/serum starvation.^14^ TRIP-Br-1 involves the propagation of cells by modulating ubiquitination of ASK1.^15^ It suppresses the transcription process of E2F by interacting with p16^INKAa^.^10^ One interesting study showed that TRIP-Br-1 actively associated in the downregulation of TIF1α/β/p300/CBP and KRIP-1 via the PHD-bromodomain of KRAB,^6^ and TRIP-Br-1 controls cell proliferation by association with the serine/threonine PP2A and holoenzyme of PP2A-AB-alphaC.^16^ Abnormal levels of TRIP-Br-1 are associated with differentiation of the neuronal cell,^17^ resistance to the drug, and apoptotic-induced programmed cell death (PCD).^18^ Collectively, the molecular studies of TRIP-Br-1 showed that it acts in a multifactorial manner with diverse mechanisms. In this study, we are deciphering the diverse function of TRIP-Br-1 as a transcriptomic and translational biomarker for human BRCA.

TRIP-Br-2 coregulates the E2F-responsive promotor region by interacting with bromodomain factors.^6^ In contrast, it may function as E2F1-TFDP1 complexes on E2F binding sites as either a coactivator or corepressor that activates or inhibits a bunch of genes that are targeted by E2Fs. It is also involved in the regulation of oxidative metabolism, thermogenesis, and adipocyte lipolysis, modulating fat storage-expressing genes.^19^ In contrast, TRIP-Br-2 significantly regulates fat metabolism by governing the β3-adrenergic receptor and hormone-sensitive lipase. Parallelly, Cogburn and his team^20^ reported that TRIP-Br-2 was significantly involved as a lipogenic transcription factor in a newly hatched embryo during the metabolic stress or fasting. It was well reported by Cheong et al.^21^ that TRIP-Br-2 potentially stabilizes the G2/M cell checkpoint by nuclear entrapment. Another study showed that TRIP-Br-2 controls the cell cycle through mitogenic signaling cascades.^7^ An elevated level of TRIP-Br-2 regulates the E2F/DP-transcriptional pathway via CDC6, cyclin-A2, DHFR, and cyclin-E in murine fibroblasts and nude mice.^22^ The overexpression of TRIP-Br-2 is correlated with a worse clinical prognosis of hepatocellular carcinoma. Collectively, these findings suggested that TRIP-Br-2 acts as a new molecular target for obesity, hyperlipidemia, insulin resistance, as well as cancers. TRIP-Br-3 plays a crucial role in cellular functions,^23^ which binds with the BIR2 domain of anti-apoptotic XIAP, activates autophagy, and inhibits apoptosis.^10^ Another study also proposed that TRIP-Br-3 is involved in cell checkpoint initiation via interphase and mitotic spindle processes and JUN oncogene-mediated cell-fate determination.^24^ Additionally, in a starved condition, TRIP-Br-3 induces apoptosis by destabilizing the XIAP protein.^25^ On the other hand, low levels of TRP-Br-3 were observed in cancer than normal cells, but TRIP-Br-3 significantly regulates antitumor activities.^25^^,^^26^ Therefore, it is known to be tumor suppressors rather than carcinogenic factors in a few cases that are downstream of the Nrf2 signaling pathway.^26^ TRIP-Br-4 is involved in some molecular signaling, including carcinogenesis.^27^ As reported by Zhang et al.^28^, TRIP-Br-4 is associated with microRNA-92a and controls prostate cancer via p53 signaling cascades. One variant of TRIP-Br-4 significantly modulates cell-cycle progression in a G1- and S-phase E2F-dependent manner.^27^ TRIP-Br-4 binds with the second subunit of the human RPA32 protein and has significantly higher activity in cancer-transformed cells.^29^ The last member of the TRIP-Br family nuclear factor TRIP-Br-5 regulates transforming growth factor (TGF)-β-mediated cardiac fibroblast activation.^30^

However, the fundamental phenomenon by which TRIP-Brs are involved in several cancers, including BRCA, has remained to be explained. The dysregulation in the expression of the TRIP-Br family and its relationship with clinicopathological, as well as prognostic values, have been partly reported in some literature. To the best of our knowledge, this is the first work that comprehensively explores the potential functions of TRIP-Br nuclear factors in carcinogenesis, clinicopathological significances, and its prognostic values in patients with BRCA. Nucleic acid DNA- and RNA-based explorations are revolutionizing with the upgraded microarray technology in the field of oncological research. In the current study, we elucidated the potential roles of TRIP-Brs, along with the thousands of expressed genes, copy number variations, mutations, correlations, and interaction in the diverse biological processes and pathways using the published data online. We have also investigated the expression of TRIP-Brs and its relationship with clinicopathological prognostic values in patients with BRCA. These studies may provide a new way to address the unclear mechanism of TRIP-Brs in BRCA, which will be highly beneficial for the management of BRCA.

## Results

### TRIP-Brs Are Actively Involved in Patients with Multiple Human Cancers, Including BRCA

**Figure 1:**
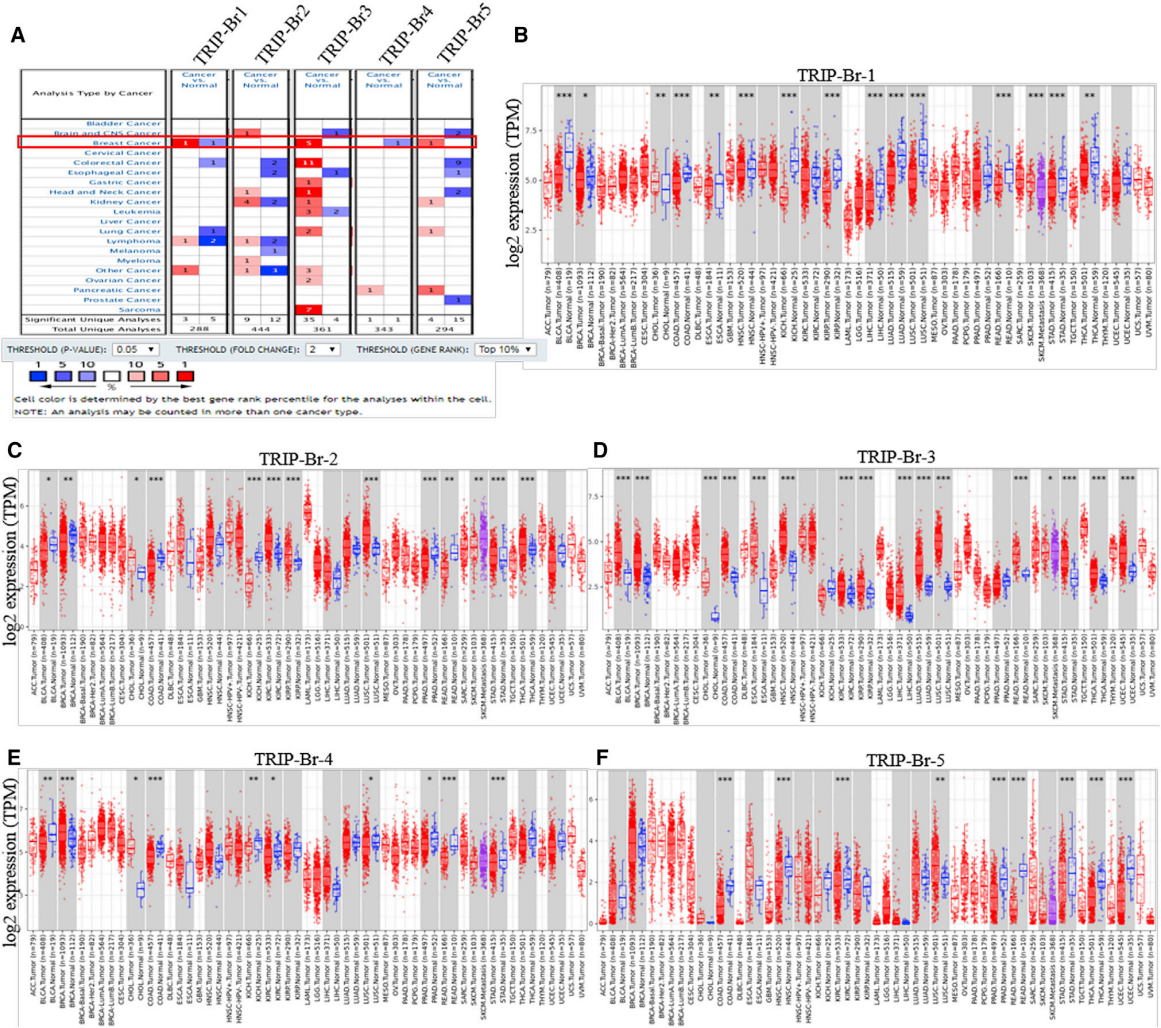
The Association of TRIP-Brs in Multiple Carcinomas Including Breast Cancer (BRCA) (A) A significant overexpression was found in most of the cancers, including BRCA patients’ tissue multiple datasets. The mRNA levels and gene rank of TRIP-Brs were also elucidated in BRCA patients’ versus normal breast tissue samples. The datasets were shown with statically significant (p < 0.01) mRNA levels in red and blue. Red, overexpression; blue, downregulated (Oncomine database). (B–F) Differential expression pattern of TRIP-Brs (statistically significant values were indicated in ∗p < 0.05, ∗∗p < 0.01, and ∗∗∗p < 0.001) in multiple carcinoma with adjacent normal tissues.

TRIP-Br factors can be seen elevated in higher mammals and are associated with many biological phenomena. We have investigated the mRNA expression of TRIP-Brs in tumor tissues with respect to their corresponding normal samples by Oncomine^31^ databases (Figure 1A). It was depicted that elevated fold changes with a higher rank of TRIP-Brs were found in most cancers, including BRCA, than normal breast tissue samples. Additionally, the transcriptomic levels of TRIP-Br-1 were significantly overexpressed in BRCA patients’ samples with respect to normal in four datasets. In Finak et al.’s dataset,^32^ the expression of TRIP-Br-1 was elevated with mRNA fold change 3.771 (t test = 20.664) in invasive BRCA tissues with respect to normal breast tissues (Table 1). The Cancer Genome Atlas (TCGA) BRCA statistics also showed higher expression with fold mRNA level 1.236 in ductal BRCA and in medullary BRCA with a fold mRNA level of 1.135. Glück et al.^33^ reported that TRIP-Br-2 was overexpressed in invasive BRCA with mRNA levels 1.380, lobular BRCA 1.164, and ductal BRCA 1.025 as compared to normal breast tissue samples. Another study by Curtis et al.^34^ found mRNA levels of TRIP-Br-3 in medullary breast carcinoma with 2.302, invasive ductal BRCA with 1.773, tubular BRCA with 1.365, and invasive BRCA with 1.727 (Table 1). Furthermore, TCGA datasets showed an elevated fold change of TRIP-Br-3 that was observed in mucinous breast carcinoma (3.060), invasive ductal BRCA (2.250), and intraductal cribriform breast adenocarcinoma (2.202). Another study by Karnoub et al.^35^ also showed a higher expression of TRIP-Br-3 in invasive ductal BRCA with 2.401 compared to normal tissue (Table 1). A higher expression of TRIP-Br-4 was also seen in invasive breast carcinoma stroma with 2.022 mRNA levels and male breast carcinoma with fold change 1.44 that was reported by Finak et al.^32^ and TCGA analysis. Nuclear factor TRIP-Br-5 was significantly upregulated in invasive ductal breast carcinoma stroma (Ma breast4 statistics, fold change = 1.812), invasive ductal breast stroma carcinoma (fold change = 2.151), and tubular breast carcinoma (Curtis et al.^34^, mRNA fold change = 1.666) with respect to normal breast tissue samples (Table 1). To validate the transcriptomic fold of TRIP-Brs in various human carcinomas, we analyzed expression of mRNA levels of TRIP-Brs using the Tumor Immune Estimation Resource (TIMER)^36^ TCGA datasets. The levels of TRIP-Brs in tumor and normal tissues have been shown in Figures 1B–1F. The TRIP-Brs significantly overexpressed in BRCA, kidney-carcinoma (KIRC), cholangiocarcinoma (CHOL), lung-adenocarcinoma (LUAD; except TRIP-Br-1), colon-adenocarcinoma (COAD), esophageal-carcinoma (ESCA), uterine-endometrial-carcinoma (UCEC), and lung squamous cell carcinoma (LUSC; except TRIP-Br-1), whereas significant downregulation of TRIP-Brs was observed in KICH (kidney chromophobe) and PRAD (prostate adenocarcinoma) as compared with normal tumor tissue samples (Figures 1B–1F).

**Table 1:** The Significant Changes of TRIP-Br Expression in Transcriptional Level between Different BRCA versus Normal Breast Tissues (Oncomine Database)

TF	Types of BRCA versus Normal Breast Tissue	Fold Change	p Value	t Test	Source and/or Reference
TRIP-Br-1	invasive breast carcinoma	3.771	1.52E−27	20.664	Finak breast statistics
	ductal breast carcinoma	1.236	0.117	1.397	TCGA
	medullary breast carcinoma	1.135	0.195	1.003	TCGA
	male breast carcinoma	1.059	0.051	1.850	TCGA
	invasive ductal and invasive lobular breast carcinoma	1.040	0.231	0.903	TCGA
TRIP-Br-2	invasive breast carcinoma	1.380	0.002	5.978	Glück breast statistics
	lobular breast carcinoma	1.164	0.223	0.815	Sorlie breast statistics
	ductal breast carcinoma	1.025	0.428	0.193	Sorlie breast statistics
TRIP-Br-3	medullary breast carcinoma	2.302	4.08E−15	12.980	Curtis breast statistics
	invasive ductal breast carcinoma	1.773	4.91E−97	34.075	Curtis breast statistics
	tubular breast carcinoma	1.365	2.56E−26	12.715	Curtis breast statistics
	invasive breast carcinoma	1.727	4.19E−7	6.838	Curtis breast statistics
	mucinous breast carcinoma	3.060	0.001	7.608	TCGA
	invasive ductal breast carcinoma	2.250	2.12E−34	17.881	TCGA
	intraductal cribriform breast adenocarcinoma	2.202	2.68E−4	10.715	TCGA
	invasive ductal breast carcinoma	1.687	0.031	2.239	Turashvili breast statistics
	invasive ductal breast carcinoma stroma	2.401	0.008	3.220	Karnoub breast statistics
	invasive ductal breast carcinoma epithelia	1.603	0.004	3.265	Ma breast4 statistics
	ductal breast carcinoma	1.679	6.58E−4	3.927	Richardson breast2 statistics
	invasive breast carcinoma	1.668	0.027	3.030	Glück breast statistics
TRIP-Br-4	intraductal cribriform breast adenocarcinoma	1.453	0.030	2.719	TCGA
	male breast carcinoma	1.444	0.038	2.539	TCGA
	invasive breast carcinoma stroma	2.022	1.91E−6	8.357	Finak breast statistics
	ductal breast carcinoma *in situ*	1.172	0.001	3.935	Curtis breast statistics
TRIP-Br-5	invasive lobular breast carcinoma	1.340	1.45E−23	14.805	TCGA
	invasive ductal and lobular breast carcinoma	1.450	0.016	5.388	TCGA
	invasive ductal breast carcinoma stroma	2.151	4.74E−4	3.883	Karnoub breast statistics
	tubular breast carcinoma	1.666	3.33E−15	8.929	Curtis breast statistics
	ductal breast carcinoma *in situ* epithelia	1.501	0.005	3.165	Ma breast4 statistics
	invasive ductal breast carcinoma stroma	1.812	0.018	2.292	Ma breast4 statistics
	invasive breast carcinoma	1.444	1.98E−4	3.634	TCGA

NA, not available; BRCA, breast cancer; TCGA, The Cancer Genome Atlas; TF, transcription factor.

### The Expression Pattern of TRIP-Brs in BRCA Cell Lines and Patient Tissues

**Figure 2:**
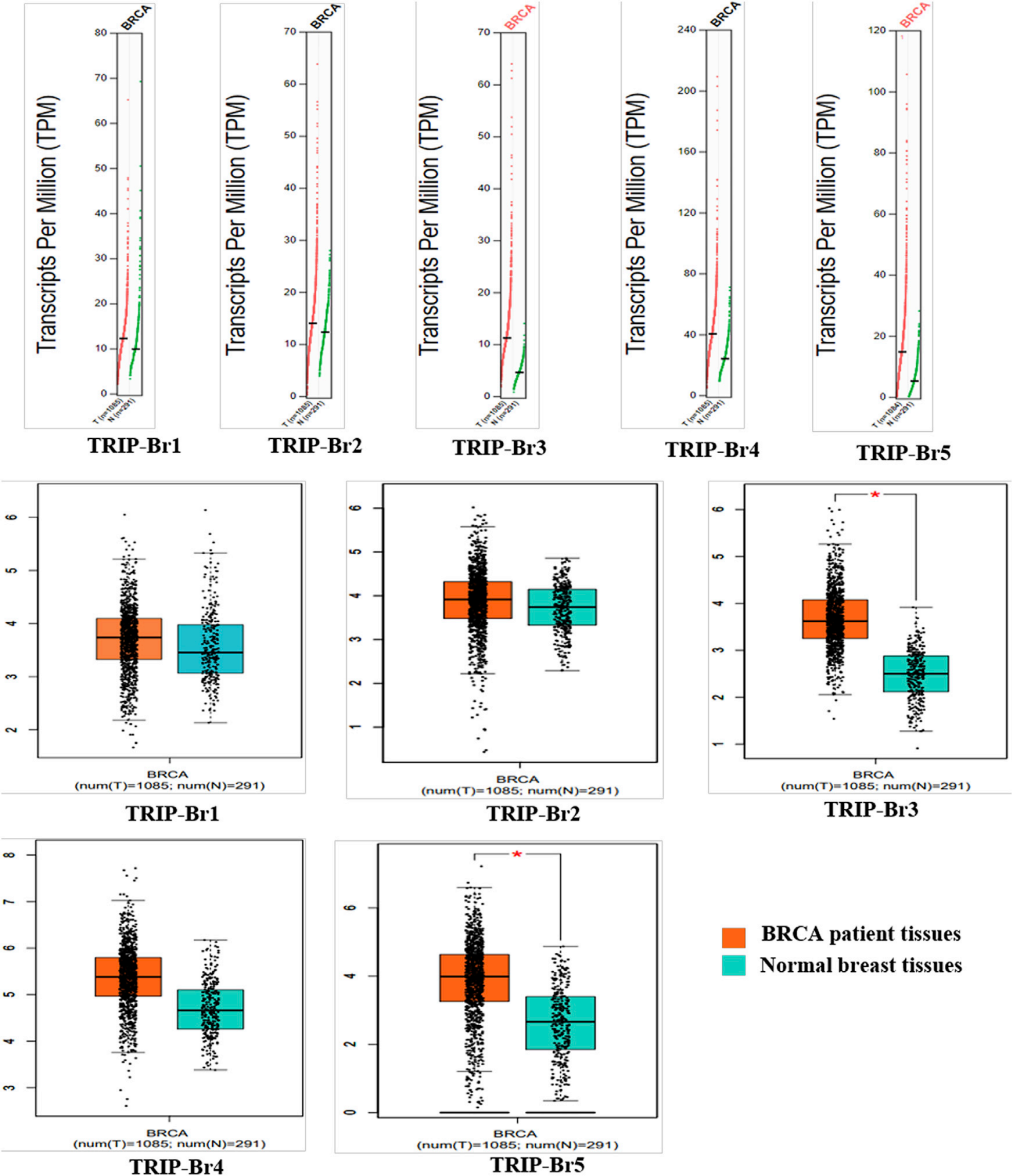
The Expression Pattern of TRIP-Brs in BRCA and Normal Tissues The GEPIA analysis dot boxplots showed the expression pattern of TRIP-Brs in BRCA tissues versus normal samples. In the graph, two groups were classified based on colors: coral = tumor group, and teal = normal tissues group. The one-way ANOVA was used to differentiate variable for calculation of expression pattern, and significant values were depicted using asterisks with patients and normal tissue samples. TPM, transcripts per million; T, tumor; N, normal.

The involvement of TRIP-Brs has been elucidated as a potential factor in several datasets of BRCA. Our aim was to confirm the key functions of TRIP-Brs in cancer patients’ tissue with respect to normal samples. The genomic profiler Gene Expression Profiling Interactive Analysis (GEPIA)^37^ is an online web server database system that contains tumor tissue samples (n = 9,736) versus normal tissue samples (n = 8,587) from the TCGA and the Genotype-Tissue Expression (GTEx) platform. Hence, we have comprehensively analyzed the mRNA levels of TRIP-Brs between normal and tumor samples. Interestingly, dot plot results showed that TRIP-Br-3 and TRIP-Br-5 were significantly elevated in patients with BRCA than normal adjacent samples. TRIP-Br-3 and TRIP-Br-5 levels were found to be enhanced in BRCA than normal corresponding samples. In addition, the dot plot analysis also displayed a higher level in BRCA cancer patients’ tissue with respect to normal tissue (Figure 2A). To verify the correlation between the transcriptional levels and BRCA progression, we also examined the expression of TRIP-Brs by box and whisker analysis; significantly higher RNA levels of TRIP-Br-3 and TRIP-Br-5 were observed in BRCA patients as compared with their counter normal tissue. TRIP-Br-3 and TRIP-Br-5 demonstrated a median relative expression in BRCA patients. However, the mRNA levels of TRIP-Br-1, TRIP-Br-2, and TRIP-Br-4 were also diverse in BRCA versus normal tissue (Figure 2B).

**Figure 3:**
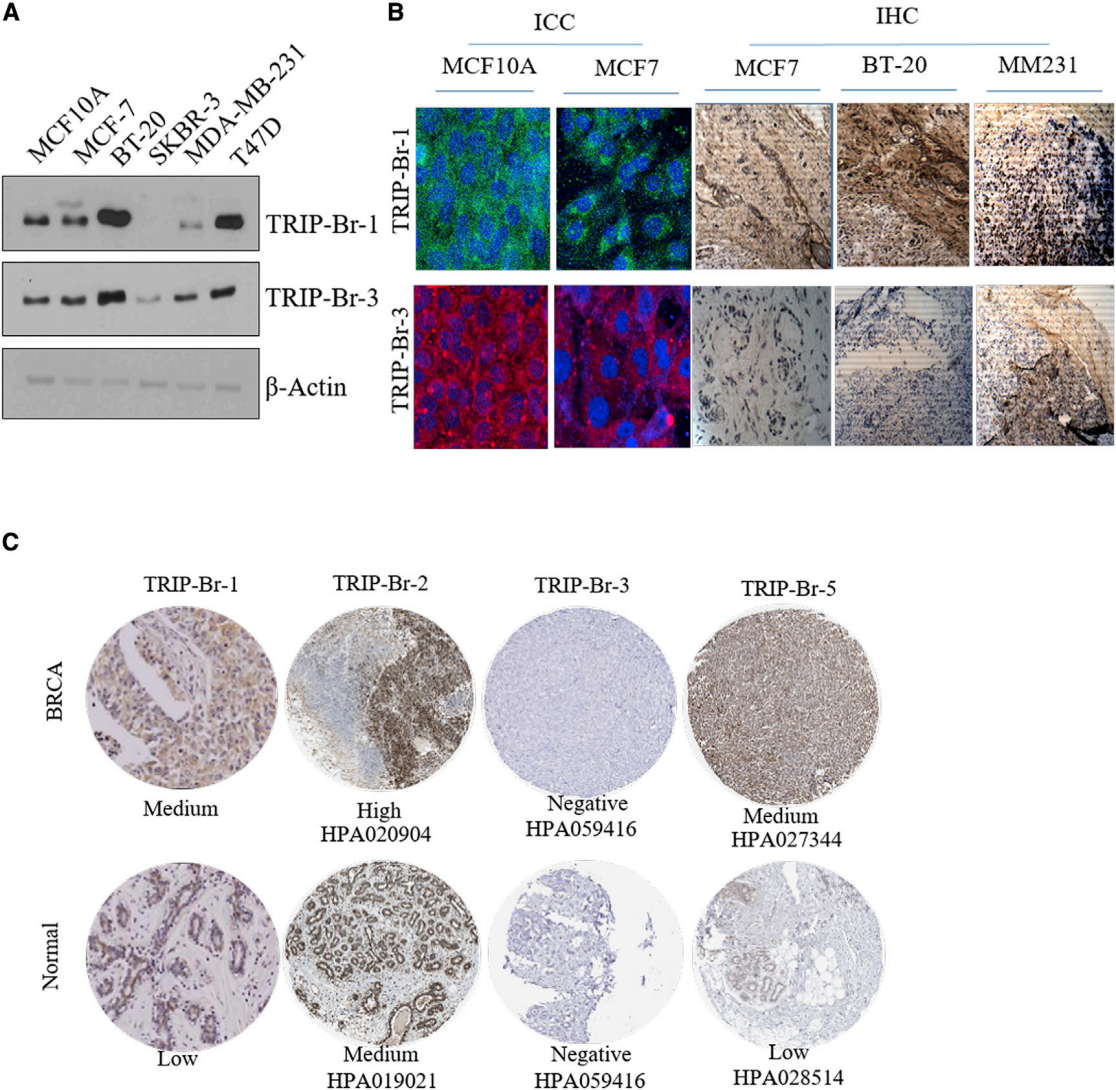
The Expression of TRIP-Brs in BRCA Cell Lines and IHC Tissues (A) Immunoblot study is showing the protein expression of TRIP-Br family members in different BRCA cell lines. (B) The immunocytochemistry and histopathological analyses of TRIP-Brs have been investigated in MCF7 versus MCF10A cells. In contrast, histopathological analysis of expression of TRIP-Br proteins in different tumor tissues from MCF7, BT-20, and MDA-MB-231 inoculated the xenograft. (C) The pathological IHC staining of TRIP-Br-1 from Jung et al.^18^ and TRIP-Br-2/3/5 of BRCA patients’ tissues versus normal tissues were procured from the Human Protein Atlas (HPA) database. However, no immunopathological expression (IHC) of TRIP-Br-4 was found in the HPA database.

In order to explore the possible prognostic and therapeutic role of TRIP-Brs in BRCA, first, we tried to investigate the protein levels in different types of BRCA cells lines. Noticeably, TRIP-Br-1/3 are showing elevated in BRCA cell lines than normal MCF10A cells (Figure 3A). In addition, it has been indicated that TRIP-Br-1 overexpressed in triple-negative BRCA (TNBC) cell lines BT-20 and T47D and was low, as well as not expressed, in MDA-MB-231 and SKBR-3 cells. The expression of TRIP-Br-3 was significantly overexpressed in MCF7, BT-20, and MDA-MB-231 cells as compared to normal MCF10A cells, and low expression was observed in SKBR-3 cells (Figure 3A). Further, we also observed its upregulation at translational level by immunocytochemistry and it showed that TRIP-Br1/3 were significantly higher in MCF7 compared to MCF10A cells (Figure 3B). Further, we validated translational upregulation of these proteins using a xenograft model of basal-A TNBC BT-20 (more aggressive and worse prognosis), luminal-B MCF7 (medium aggression), and basal and metastatic MDA-MB-231 cells. Interestingly, the expression pattern of TRIP-Brs in xenograft immunohistochemistry (IHC) was varied from each other. TRIP-Br-1 exhibited medium and high staining, whereas negative or very low staining of TRIP-Br-3 was observed in all three xenograft tissue (Figure 3B). These findings suggested that higher levels of TRIP-Brs in cell lines and xenograft tumor tissues of BRCA provoked the query of whether overexpression of TRIP-Brs contribute in initiation and progression of BRCA or not. Therefore, we aimed to investigate the translational protein fold of TRIP-Brs in the human BRCA-associated tumor tissues by analyzing of the Human Protein Atlas (HPA) database and previous reported literature.^38^^,^^39^ We found medium expression of TRIP-Br-1/5 as compared to normal samples in which low expression was noticed. In addition, TRIP-Br-2 was also significantly overexpressed in BRCA with respect to normal tissues IHC (Figure 3C). However, the lack of expression or meager levels of TRIP-Br-3 were observed in both tumor and normal samples (Figure 3C). Collectively, current findings indicated the transcriptomic and proteomic levels of TRIP-Brs, which were highly associated in BRCA.

### TRIP-Br Expression Status within Molecular Subtypes and Stages of BRCA

**Figure 4:**
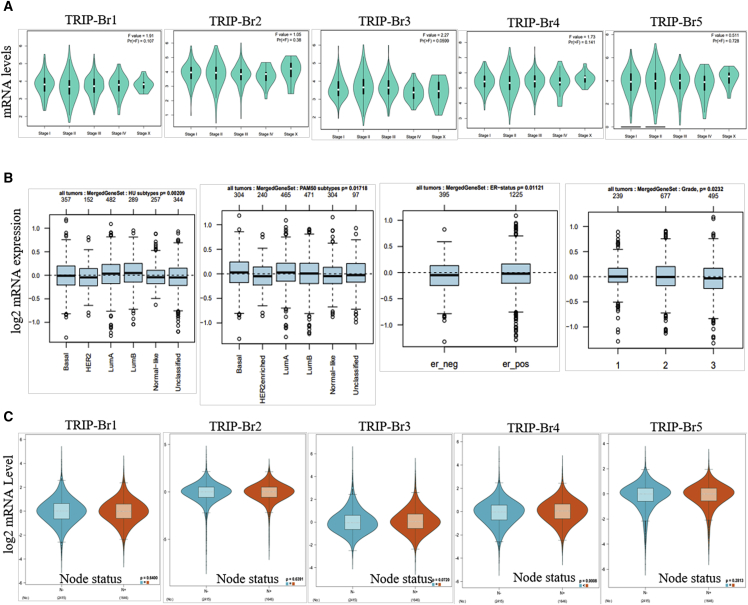
The TRIP-Brs in Different Stages, Subtypes, and Nodes of the BRCA Tumor (A) Violin plots represented the expression of TRIP-Brs in stage I∼stage IV of BRCA (GEPIA database). (B) The log2 mRNA fold of TRIP-Brs in breast microarray datasets (n = 1,881 samples) containing tumor grade/ER^+^/ER^−^ BRCA patients samples (GOBO datasets). (C) The association between log2 mRNA expression of TRIP-Brs and lymph node status in patients with BRCA.

**Figure 5:**
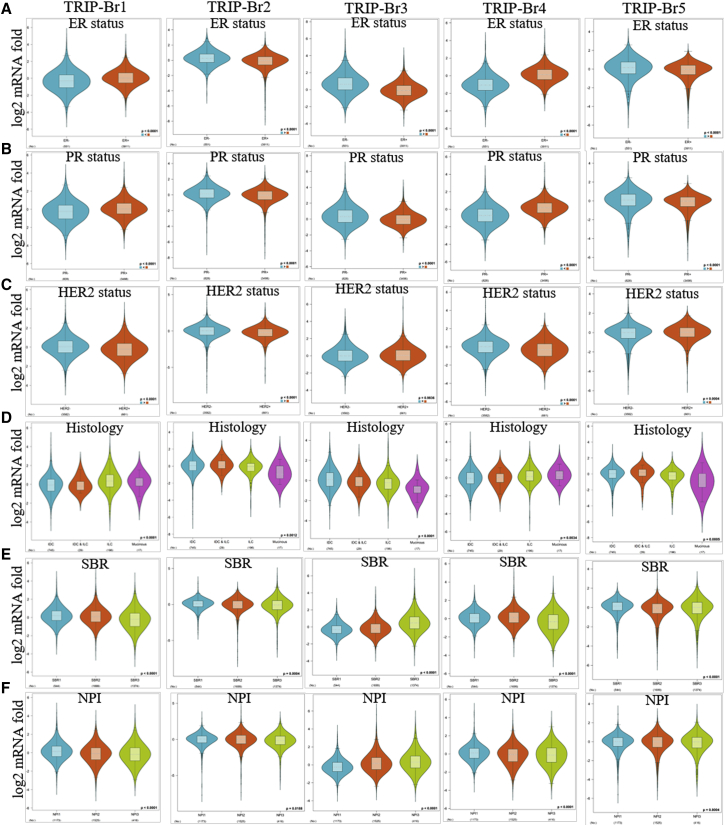
The Association between TRIP-Br Expression and Clinical Features of Patients with BRCA (A–F) The log2 expression of TRIP-Brs, especially in ER (A)/PR (B)/HER2 (C) enriched (A–C), histology (D), SBR (E), and NPI (F) in patients with BRCA. NPI, Nottingham Prognostic Index; it is a clinicopathological parameter that derives from lymph node, tumor size, and grade. SBR, Scarff-Bloom-Richardson grade; SBR is a grading system that defines breast tumor grades based on tubule formation, mitotic counts, and nuclear pleomorphism.

We found that TRIP-Brs were overexpressed in BRCA and play a crucial role in its progression. Herein, we further aimed to explore the expression pattern of TRIP-Brs in several clinical features, molecular subtypes, and stages within BRCA. The expression pattern of TRIP-Br-1/3/4 was shown variable in different stages (stage I to stage IV) of BRCA patients, and it indicates that these factors participated in the development of tumorigenesis at different stages (Figure 4A). To gain insight into BRCA progression, it was necessary to throw light on the association among TRIP-Br expression, molecular subtypes, and clinical features. The expression of TRIP-Brs was significantly higher in basal, luminal-A/B clinical subtypes, enriched ER^+^, and grade1/2 as compared to ER in the Gene Expression-Based Outcome for Breast Cancer Online (GOBO) database (Figure 4B). GOBO^40^ is a gene-signature, user-friendly tool that contains 1,881 breast tumor samples. Next, we systematically analyzed the expression of TRIP-Brs in RNA sequencing (RNA-seq) datasets (TCGA, n = 1,034; Gene Expression Omnibus [GEO]: GSE81540, n = 3,678) of different subtype of BRCA by using the Breast Cancer Gene-Expression Miner (bc-GenExMiner) version (v.)4.3 database.^41^ Higher levels of TRIP-Br-1/4 were observed in ER^+^- and PR^+^-enriched BRCA patients, whereas significantly higher TRIP-Br-2/3/5 were detected in the ER^−^/PR^−^ subtype of BRCA patients. Surprisingly, most TRIP-Brs were found to be overexpressed in HER2^negative^-enriched BRCA patients, and TRIP-Brs were not significantly associated with lymph nodes, except TRIP-Br-4 was significantly overexpressed in lymph node-containing BRCA patients (Figures 4C and 5A–5C). Histological analysis revealed that the expression of TRIP-Brs was varied in different types of BRCA: TRIP-Br-1/4 was overexpressed in invasive lobular carcinoma (ILC) and mucinous; TRIP-Br-2/5 was found to be elevated in invasive ductal carcinoma (IDC)/ILC, whereas TRIP-Br-3 was moderately expressed in IDC (Figure 5D). Histological tumor grade is an independent prognostic factor in specific subgroups for the management of BRCA patients.^42^ Intriguingly, we found that in the relationship between TRIP-Brs and histological tumor grades, higher levels of TRIP-Br-1/2/4/5 were observed in Scarff-Bloom-Richardson-1/2 (SBR-1/2) and higher Nottingham Prognostic Index-1/2 (NPI-1/2) grade. However, TRIP-Br-3 was only overexpressed in SBR-3 and NPI-3 subgrades of BRCA tumors (Figures 5E and 5F).

**Figure 6:**
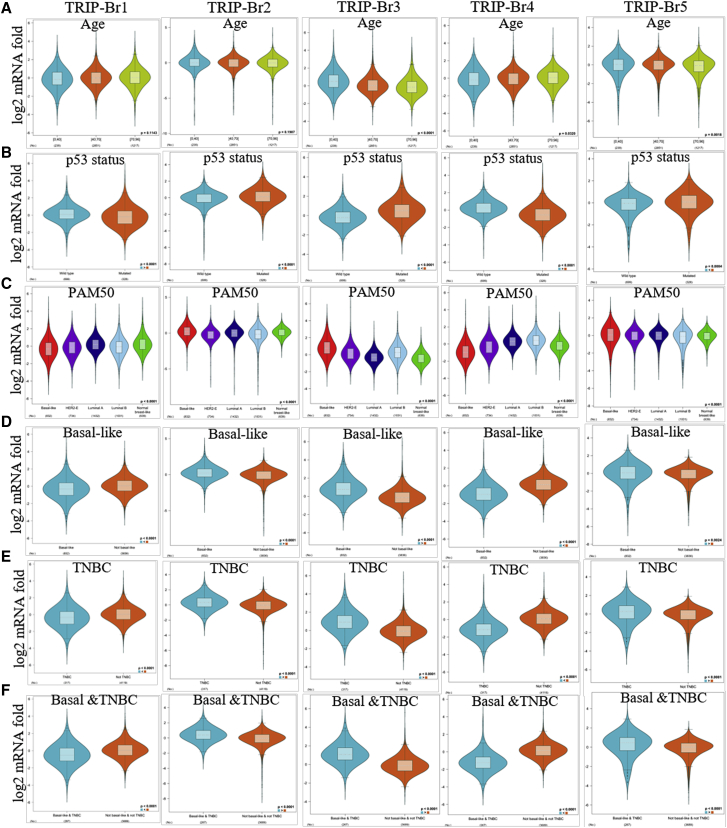
The Expression Pattern of TRIP-Brs in Diverse Clinical Subtypes of Patients with BRCA (A–F) The log2 expression pattern of TRIP-Brs was depicted in age (A), p53 status (B), PAM50 (C), basal like (D), TNBC (E), and basal and TNBC status (F) in patients with BRCA. PAM50, subtypes of BRCA.

To validate the involvement of TRIP-Brs in subtypes of BRCA that linked with other clinical features, we studied the expression pattern of TRIP-Brs, and their diverse expression was found in different aged subgroups. TRIP-Br-1/2 were not significant in different ages of BRCA patients; however, TRIP-Br-3 expression was higher in under 0 to 50 years, and TRIP-Br-4/5 were highly observed in patients over 40 to 96 years old (Figure 6A). Genomic alteration of tumor-suppressor p53 resulted in a clinical outcome in patients with BRCA.^43^ In this study, we found that expression of TRIP-Br-2/3/5 was elevated in the p53-mutated patients than p53 wild type, and it has been implicated that these factors were associated with a mutation of a p53-mediated outcome of BRCA patients (Figure 6B). TRIP-Brs were found higher in the luminal-A/B (LumA/B) subtype of BRCA (except TRIP-Br-3) in PAM50, which was consistent with GOBO analysis (Figure 6C). Higher expression of TRIP-Br-2/3/5 was found in the basal-like subtype of BRCA. Additionally, TRIP-Br-2/3/5 overexpression (except TRIP-Br-1/4) was observed in TNBC and the basal like-TNBC subtype of BRCA patients (Figures 6D–6F).

### Prognostic and Clinicopathological Significance of TRIP-Brs in BRCA

**Figure 7:**
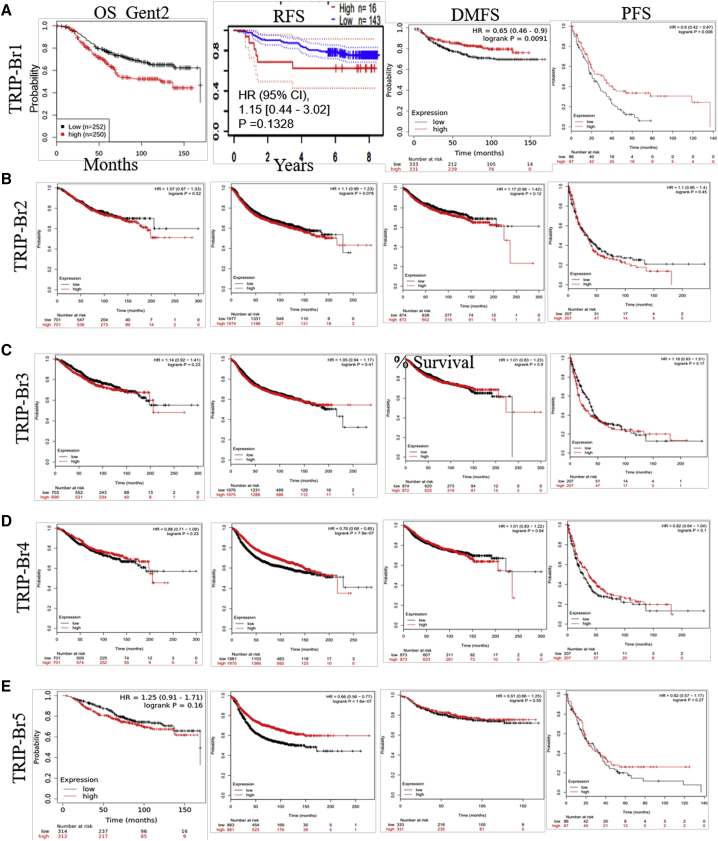
The Prognostic Value of Nuclear Factor TRIP-Brs in BRCA Patients (A) Prognostic value of TRIP-Br-1 showed overall survival (OS; GENT2 datasets analysis), relapse-free survival (RFS; PrognoScan datasets), distant metastasis-free survival (DMFS), and PFS for TRIP-Br-1 in BRCA. (B–E) The prognostic values for each TRIP-Br were analyzed using KM plotter based on their median expression in BRCA patients. The Kaplan-Meier (KM) survival curve showed OS, RFS, DMFS, and disease-free survival (DFS) in low and higher TRIP-Br-expressing patient groups using KM plotter of GEO, European Genome-Phenome Archive (EGA), and TCGA datasets.

To explore the correlation between transcriptomic levels of TRIP-Brs and prognosis of patients with BRCA, we used the Kaplan-Meier (KM) Plotter interactive web tool (https://kmplot.com/analysis/index.php?p=service&cancer=breast).^44^^,^^45^ In this line, we examined the independent prognosis for each TRIP-Br based on mRNA fold, especially in order to determine overall survival (OS), relapse-free survival (RFS), distant metastasis-free survival (DMFS), and progression-free survival (PFS) in BRCA patients. We found that TRIP-Brs were significantly overexpressed in patients’ tissues of BRCA than normal tissue samples, which lead to regulate survival of patients (Figures 7A–7E). The patients’ survival curves were shown in Figure 7 using a log rank test that evoked increased levels of TRIP-Br-1 (Gene Expression Database of Normal and Tumor Tissues [GENT2] datasets, p = 0.001) and TRIP-Br-5 (95% confidence interval [CI] = 0.57–1.07, hazard ratio [HR] = 0.78, p = 0.13), which may be significantly correlated with the worse and shorter OS of patients with BRCA (Figure 7A). The TRIP-Br-2 (95% CI = 0.87–1.33, HR = 1.07, p = 0.52), TRIP-Br-3 (95% CI = 0.92–1.41, HR = 1.44, p = 0.23), and TRIP-Br-4 (95% CI = 0.71–1.09, HR = 0.88, p = 0.23) associated with higher OS in the starting 100 days, and lower prognostic values (OS) were noticed post-100 days (Figures 7B–7D). Additionally, KM log rank analysis showed the higher TRIP-Br-2/3 mRNA levels and decreased TRIP-Br-1/5 expression, which were significantly (p < 0.05) correlated with PFS, RFS, and DMFS in breast carcinoma patients (Figures 7A–7E). Further, higher mRNA levels of TRIP-Br-4 (95% CI = 0.99–4.37, HR = 2.08, p = 0.048) were statistically linked with shorter RFS, PFS, and DMFS of patients with BRCA (Figure 7D). These results indicated that TRIP-Brs play an important function in tumor-bearing patients with significant prognostic values, and they may be functioned as valuable biomarkers to predict the survival of BRCA patients. In the Cox hazard model, univariate analysis also showed that overexpression of TRIP-Brs was significantly associated with low survival and tumor stage III (TRIP-Br-1/HR = 3.893; TRIP-Br-2/HR = 4.050; TRIP-Br-3/HR = 3.888; TRIP-Br-4/HR = 3.868; TRIP-Br-5/HR = 3.774, p < 0.05) and stage IV (TRIP-Br-1/HR = 13.741; TRIP-Br-2/HR = 11.437; TRIP-Br-3/HR = 11.106; TRIP-Br-4/HR = 10.548; TRIP-Br-5/HR = 11.491, p < 0.05) in patients with BRCA (Table S1). However, there was no significant OS was observed in N stage in patients with higher TRIP-Br-expressing cohorts except TRIP-Br-5 (HR = 1.87 [1.08–3.22, p < 0.0222]) (Figure 7E; Table S1). Collectively, these findings indicated that the transcriptional levels of TRIP-Brs are independent survival factors in patients with BRCA.

The Cox proportional hazards regression model with OS, PFS, and RFS in BRCA patients is depicted in Figures S1–S3. The mRNA levels of TRIP-Br-1 (HR = 1.0, I^2^ = 0%, τ^2^ = 0, p = 0.57) and TRIP-Br-2 (HR = 1.0, I^2^ = 21%, τ^2^ = 0.0001, p = 0.06) were not significantly correlated in BRCA patients with OS (Figure S1). However, some patients were found to have increased TRIP-Brs associated with both low and high risk of BRCA patients. Interestingly, an elevated mRNA level of TRIP-Br-1 was correlated with a lower risk of death in term of PFS (HR = 0.72, I^2^ = 48%, τ^2^ = 0.1653, p = 0.11) and RFS (HR = 1.0, I^2^ = 62%, τ^2^ = 0.0001, p = 0.02). Overexpression of TRIP-Br-2 (HR = 1.24, 95% CI = 0.99–1.53, I^2^ = 0%, τ^2^ = 0.0, p = 0.87) was observed in BRCA patients and was significantly associated with high risk and worse PFS (Figure S1). Similarly, no significant association among mRNA expression of TRIP-Br-3 (HR = 1.0, I^2^ = 44%, τ^2^ = 0.0001, p = 0.01), TRIP-Br-4 (HR = 1.0, I^2^ = 37%, τ^2^ = 0.0001, p = 0.01), TRIP-Br-5 (HR = 1.0, I^2^ = 0%, τ^2^ = 0.0, p = 0.62), and the OS in all BRCA cancer patients (Figures S1–S3) was identified. A strong association was also observed in the increased TRIP-Br-3 (PFS, HR = 1.28, 95% CI = 0.82–1.88, I^2^ = 18%, τ^2^ = 0.0563, p = 0.29) with worse PFS, whereas enhanced in TRIP-Br-5 (PFS, HR = 0.94, 95% CI = 0.85–1.04, I^2^ = 23%, τ^2^ = 0.0098, p = 0.18), and it was significantly linked with better prognosis in all BRCA patients (Figure S3). In addition, mRNA levels of TRIP-Br-3 (RFS, HR = 1.0, I^2^ = 85%, τ^2^ = 0.0001, p = 0.01), TRIP-Br-4 (RFS, HR = 1.0, I^2^ = 53%, τ^2^ = 0.0001, p = 0.04), and TRIP-Br-5 (RFS, HR = 1.0, I^2^ = 64%, τ^2^ = 0.0001, p = 0.01) were not statistically significant with RFS in all cohorts of BRCA patients (Figure S3). Our study distinctly showed that overexpression of TRIP-Brs was significantly associated with prognostic values of OS, PFS, and RFS of patients with BRCA. Therefore, we assumed that TRIP-Brs are playing as key regulators in initiation, recurrence, and metastasis of BRCA.

### Mutational Changes in TRIP-Brs Modulate the Clinicopathological Prognosis of Patients with BRCA

**Figure 8:**
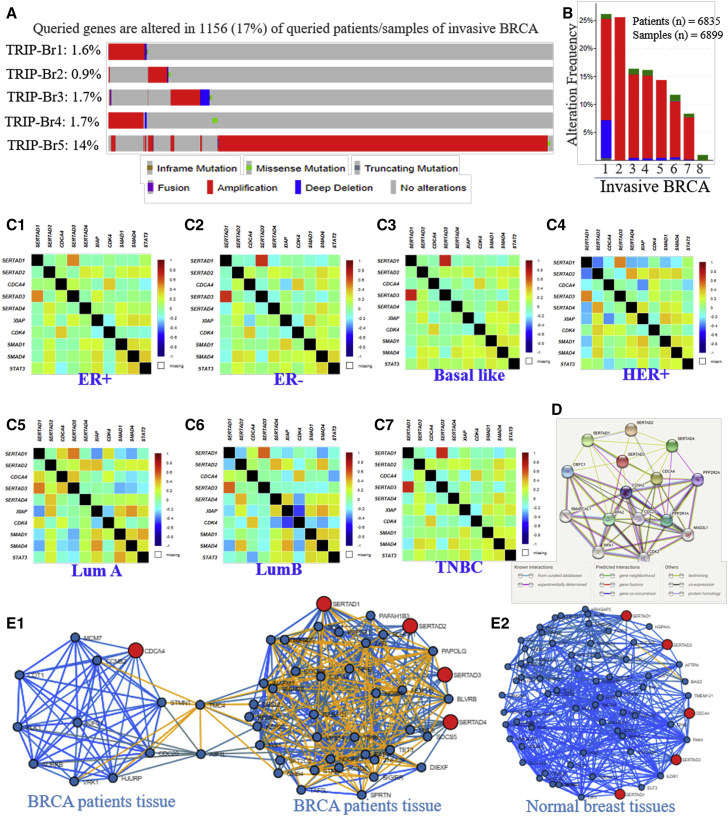
OncoPrint Genomic Alterations, Correlations, and Protein-Protein Interaction of TRIP-Br-Associated Genes in BRCA and Normal Breast Mammary Tissues (A and B) The OncoPrint is showing mutation and expression analyses in BRCA (cBioPortal). (C1–C7) Pearson correlation between TRIP-Brs and associated genes in different types of BRCAs. (D) Protein-protein interaction among TRIP-Brs (STRING protein interaction analysis). (E1 and E2) Tissue- and cancer-specific biological network (TCSBN). The positive and negative Pearson correlations were calculated (−log10 p value) using the TCSBN database. The correlation network is depicting positive with blue and negative with yellow color and queried genes in red color.

The major aims of the current study are to elucidate genomic aberration in TRIP-Brs that could affect patients’ survival, outcome, and prognosis in BRCA. Interestingly, in 1,281 BRCA patients, we have explained mutational changes on TRIP-Brs using the cBioPortal^46^ cancer database tool (http://www.cbioportal.org/index.do?), and it was shown that TRIP-Brs were mutated in multiple carcinomas (Figure S4). We found that TRIP-Brs were mutated in 1,345 tissue specimens of 1,281 metastatic and invasive BRCA patients. In addition, the alteration of TRIP-Br-1 = 2.2% (29/1,336), TRIP-Br-2 = 1.4% (19/1,336), TRIP-Br-3 = 3% (40/1,336), TRIP-Br-4 = 2.2% (30/1,336), and TRIP-Br-5 = 13% (170/1,336) was detected in patients with BRCA (Figures 8A and 8B). The mutational changes, including amplification, truncating mutation, missense mutation, and deep deletion, were found in 242 samples (18%) of 235 patients out of a 1,281 (1,336 samples) queried BRCA patient cohort (Figures 8A and S4). It was well reported that mutation and expression of any gene were directly associated with tumor progression, as well as the survival of patients with cancers, including BRCA. In the current investigation, we aimed to elaborate on the consequence of mutations in the TRIP-Br family for modulation in OS, disease-free survival (DFS), PFS, and disease-specific survival (DSS) of patients with BRCA. The metastatic BRCA (TCGA Nature, 2012) and BRCA TCGA Pan-Cancer studies showed that mutations of TRIP-Brs resulted in 160.9 months’ survival and low prognosis as compared to 280.9 months in the unaltered group (Figure S5). It showed that alteration in TRIP-Brs (log rank test, p = 0.0188) significantly decreased the OS of BRCA patients (Figure S5). The median months’ disease-free survival curves showed significant (log rank test, p = 0.0812) lower survival (altered = 32 months; without altered = 167 months) that was observed in a TRIP-Br-altered patients’ group as compared to cases without alterations. Further, median PFS of patients with BRCA showed significantly (log rank test, p = 6.522e–3) worse and shorten survival (altered = 160.9 months; without altered = 281.2 months) as compared to without altered patients. In addition, the patients with TRIP-Br mutations have secured 58.11 median months’ DSS (log rank test, p = 0.0048) than 60.82% in the without-altered patient cohort. Here, we confirmed that the effect of the mutation in TRIP-Brs significantly affected patients’ outcome and prognosis of BRCA (Figure S5). Therefore, we need to explore the interaction, correlation, and cellular processes of TRIP-Brs with vital signaling pathways in breast carcinogenesis.

### The Correlation between Altered TRIP-Brs and Neighbor Genes in BRCA Patients

To investigate the correlation of TRIP-Brs with each other in BRCA using bc-GenExMiner v.3.0,^41^ the correlation coefficients were determined with a p value for TRIP-Brs in each pair, and they indicated significant and positive correlation between TRIP-Brs, along with other associated factors: TRIP-Br-1 with TRIP-Br-4 and STAT3; TRIP-Br-2 with TRIP-Br-5, SMAD1, and SMAD4; TRIP-Br-3 with CDK4; and TRIP-Br-4 with TRIP-Br-1 and XIAP in both ER^positive^/^negative^ BRCA (Figures 8C1 and 8C2). In basal-like and HER^+^ BRCA, TRIP-Br-1 with TRIP-Br-4 and TRIP-Br-2 with TRIP-Br-5, XIAP, CDK4, SMAD1, and SMAD4 are positively regulated (Figures 8C3 and 8C4). In addition, TRIP-Br-1 was positively correlated with TRIP-Br-3, TRIP-Br-4, and CDK4; TRIP-Br-2 with TRIP-Br-5 and XIAP; TRIP-Br-3 with TRIP-Br-1, TRIP-Br-4, and CDK4; TRIP-Br-4 with TRIP-Br-1 and TRIP-Br-3; and TRIP-Br-5 with TRIP-Br-2 in luminal-A BRCA, whereas TRIP-Br-1 was negatively correlated with XIAP and SMAD4 in both luminal BRCA (Figures 8C5 and 8C6) and interacted with each other in protein level (Figure 8D). TRIP-Br-2 negatively correlated with TRIP-Br-1 and TRIP-Br-3 with XIAP and STAT3 in HER^+^ BRCA (Figures 8C4). Subsequently, we further explored tissue- and cancer-specific biological network (TCSBN) correlation analysis in BRCA tissues versus normal tissue to address correlation; results suggested that TRIP-Br-1 positively regulates with INAFM1 (Corr = 0.564, p = 3.253e–91), MYL6 (Corr = 0.570, p = 1.843e–93), RABAC1 (Corr = 0.573, p = 5.016e–95), JOSD2 (Corr = 0.615, p = 6.299e–113), and NOSIP (Corr = 0.565, p = 1.136e–91), whereas it significantly regulates (negative) to PAPOLG (Corr = −0.376, p = 2.071e–37), FBXO11 (Corr = −0.431, p = 9.269e–50), and USP34 (Corr = −0.400, p = 1.141e–42) (Figures 8E1). Similarly, TRIP-Br-2 significantly, positively regulates with USP34 (Corr = 0.492, p = 1.842e–66), FBXO11 Corr = 0.538, p = 7.400e–82, STRN (Corr = 0.541, p = 9.708e–83), and TET3 (Corr = 0.519, p = 5.047e–75) in BRCA. TRIP-Br-3 positively correlated with CDT1 (Corr = 0.600, p = 3.630e–106), PLK1 (Corr = 0.575, p = 1.242e–95), MCM7 (Corr = 0.575, p = 1.742e–95), KIFC1 (Corr = 0.618, p = 2.411e–114), AURKB (Corr = 0.577, p = 2.079e–96), and VRK1 (Corr = 0.611, p = 7.056e–111). TRIP-Br-4 was positively associated with TRIP-Br-1 (Corr = 0.513, p = 2.412e–73), PAFAH1B3 (Corr = 0.513, p = 4.155e–73), RAB4B (Corr = 0.551, p = 3.090e–86), and BLVRB (Corr = 0.574, p = 3.149e–95). Apart from that, TCSBN depicted that TRIP-Br-5 was positively correlated with TIPRL (Corr = 0.444, p = 4.949e–53), SFT2D2 (Corr = 0.460, p = 2.447e–57), and DIEXF (Corr = 0.474, p = 3.581e–61) in BRCA (Figures 8E1; Table S2). We also elucidated the correlation of TRIP-Brs in normal breast mammary tissue. TRIP-Br-1, TRIP-Br-2, TRIP-Br-3, TRIP-Br-4, and TRIP-Br-5 were positively correlated with BCL3 (Corr = 0.532, p = 5.091e–17), PSMC4 (Corr = 0.542, p = 9.352e–18), PNO1 (Corr = 0.552, p = 1.884e–18), UBE2S (Corr = 0.588, p = 2.701e–21), TBC1D22B (Corr = 0.594, p = 8.055e–22), AFTPH (Corr = 0.685, p = 5.799e–31), HSPA4L (Corr = 0.621, p = 3.424e–24), HIST3H2A (Corr = 0.620, p = 4.319e–24), HES1 (Corr = 0.665, p = 1.139e–28), and PAF1(Corr = 0.582, p = 8.570e–21), whereas negative correlation was not observed in TRIP-Brs in breast mammary tissues (Figures 8E2; Table S3). Additionally, Coexpedia GeneSet analysis also showed that TRIP-Brs were coexpressed with several genes and modulated numerous biological processes in multiple diseases, including BRCA (Tables S4–S6). Collectively, these findings pointed that TRIP-Brs played crucial roles in the carcinogenesis of BRCA by regulating several genes.

### Functional Annotation, GO Enrichment, and Signaling Pathways in Mutated TRIP-Brs and Frequently Altered Genes in BRCA Patients

**Figure 9:**
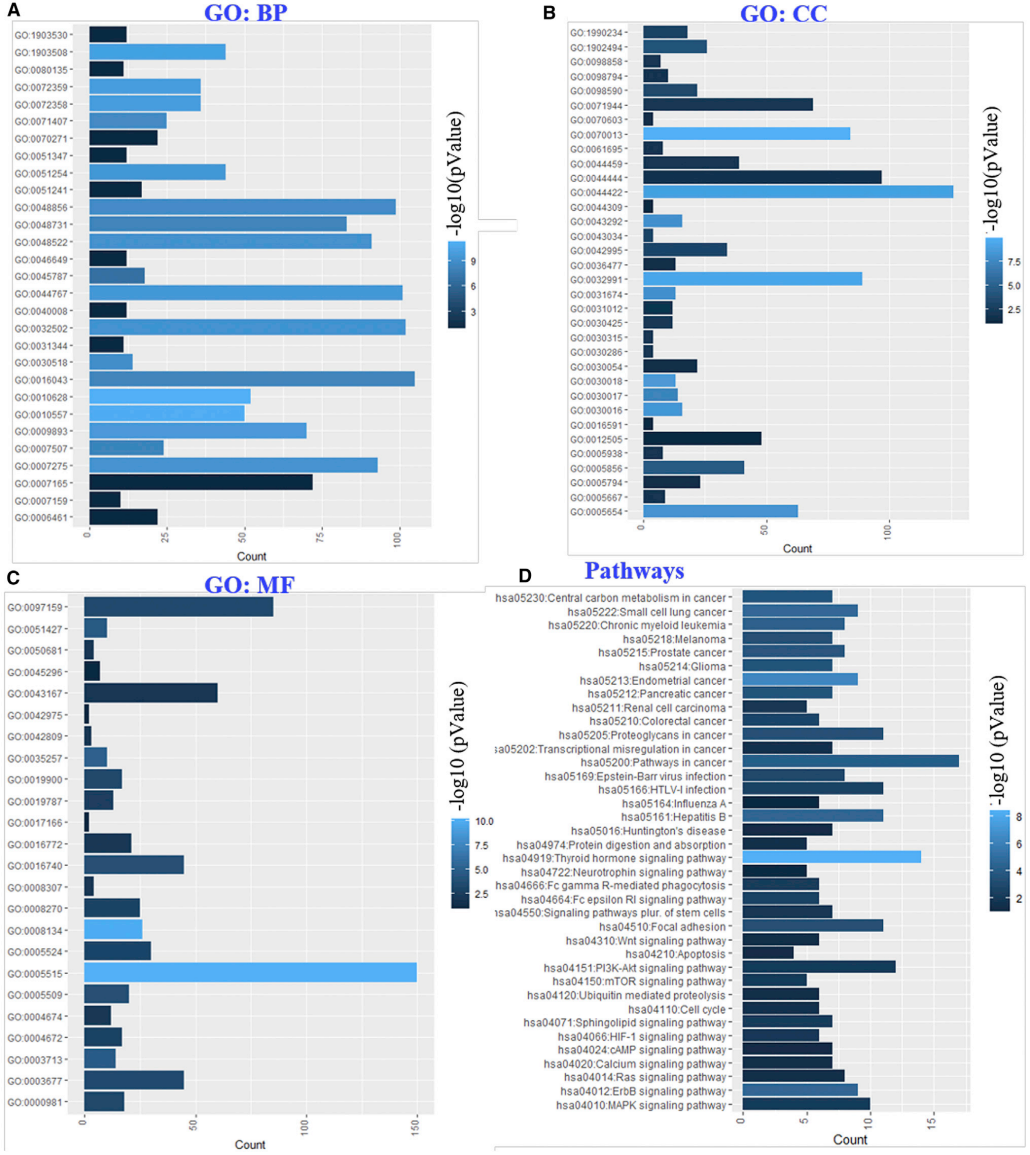
The Key Role of TRIP-Brs with Altered Neighbor Genes in Diverse Processes and Signaling Pathways (A–C) The functional gene enrichment analyzed using the DAVID tool. Three types of gene ontology: (A) biological process (GO_BP), (B) cellular components (GO_CC), and (C) molecular functions (GO_MF) have been investigated in BRCA patients’ samples. (D) Additionally, we also analyzed some significantly associated cellular pathways using KEGG analysis.

The functional role of TRIP-Brs and significantly altered genes was demonstrated by investigating their associated pathways using the KEGG (Kyoto Encyclopedia of Genes and Genomes) database system and Gene Ontology (GO) terms using the Database for Annotation, Visualization and Integrated Discovery (DAVID) database and multicluster functional gene-enrichment analyses using ToppCluster. Interestingly, enrichment analysis of GOs showed the potential functions of TRIP-Br mutations and associated genes with three types of parameters: GO_biological processes (GO_BP), GO_cellular components (GO_CC), and GO_molecular functions (GO_MF). The GO analyses revealed positive regulation of gene expression (GO: 0010628), macromolecule biosynthetic process (GO: 0010557), and cell cycle (GO: 0045787). Additionally, it has been shown that GO terms nucleic acid-templated transcription (GO: 1903508), cardiovascular system development (GO: 0072358), positive regulation of metabolic process (GO: 0009893), single-organism developmental process (GO: 0044767), intracellular steroid hormone receptor signaling pathway (GO: 0030518), anatomical structure development (GO: 0048856), and leukocyte cell-cell adhesion (GO: 0007159) were significantly regulated by TRIP-Br alterations in breast carcinoma datasets (Figure 9A). In GO_CC analysis, we observed that intracellular organelle lumen (term GO: 0070013), macromolecular complex (GO: 0032991), organelle part (GO: 0044422), Z disc (GO: 0030018), myofibril (GO: 0030016), nucleoplasm (GO: 0005654), I band (GO: 0031674), cell projection (GO: 0042995), costamere (GO: 0043034), dendrite (GO: 0030425), SWI/SNF superfamily-type complex (GO: 0070603), Golgi apparatus (GO: 0005794), endomembrane system (GO: 0012505), and neuron spine (GO: 0044309), as well as DNA-directed RNA polymerase II and holoenzyme (GO: 0016591), were also significantly controlled by alteration of these TRIP-Brs (Figure 9B). The GO_MF analyses showed that protein binding (GO: 0005515), transcription factor binding (GO: 0008134), nuclear hormone receptor binding (GO: 0035257), calcium ion binding (GO: 0005509), actin filament binding (GO: 0051015), RNA polymerase II transcription factor activity, sequence-specific DNA binding (GO: 0000981), ATP binding (GO: 0005524), androgen receptor binding (GO: 0050681), vitamin D receptor binding (GO: 0042809), cadherin binding (GO: 0045296), and transmembrane receptor protein tyrosine kinase adaptor activity (GO: 0005068) were regulated by a mutation in the TRIP-Br family in BRCA patients (Figure 9C).

The analysis of KEGG pathways showed multiple pathways that are associated with alterations of TRIP-Brs and frequently altered neighbor genes; more than 30 cellular pathways are linked to the mutations of TRIP-Brs in patients with BRCA. Among these signaling cascades, hsa05230:central carbon metabolism in cancer; hsa05213:endometrial cancer; hsa04919:thyroid hormone pathway; hsa05222:small cell lung cancer; hsa04010:mitogen-activated protein kinase (MAPK) cellular pathway; hsa04012:ErbB pathway; hsa05161:hepatitis B; hsa05220:chronic myeloid leukemia; hsa05214:glioma; hsa04151:phosphatidylinositol 3-kinase (PI3K)-Akt signaling pathway, ubiquitin-mediated proteolysis pathways; hsa05202:transcriptional misregulation in cancer; hsa04024:cyclic AMP (cAMP) signaling cascades; hsa04110:cell cycle; and hsa04722:neurotrophin signaling pathway, as well as hsa04020:calcium signaling pathways, were significantly associated with the alteration in TRIP-Brs in BRCA (Figure 9D). The extensive study of the cellular pathways, interaction, and significant GOs of associated genes with a mutation of TRIP-Brs in BRCA was also conducted using ToppCluster^47^ and cBioPortal tools.^46^ Indeed, the mutational changes in TRIP-Brs have directly interacted with BRCA_cell cycle, BRCA_TP53-pathway, and BRCA_RTK-RAS-PI(3)K pathway by modulating CCNE1 (3.7%), ATM (4.6%), RB1 (6.1%), TP53 (34.9%), ERBB2 (15.2%), PIK3CA (35.3%), and CCND1 (17.9%) genes via regulation of cell cycle, apoptosis, and cells proliferation (Figure S6). Additionally, ToppCluster analysis showed that TRIP-Brs interacted with TFDP1, ADCY1, CREBBP, E2F1, XIAP, KAT2B, and TRIM28 in BRCA. It also distinctly showed that TRIP-Brs contributed in the regulation of cell cycle and growth at the G1/S phase and process of memory by the diverse mechanism as HTLV-1 infection, pathways in cancer, transcription and translation process, pre-NOTCH pathway, intrinsic apoptosis pathway, small lung carcinoma, and hepatitis B (Figure S6). These findings designated TRIP-Br function as a key regulator in the diverse cellular program.

### Correlation of TRIP-Br Expression with Tumor Immune-Infiltration Levels in BRCA Patient Datasets

**Figure 10:**
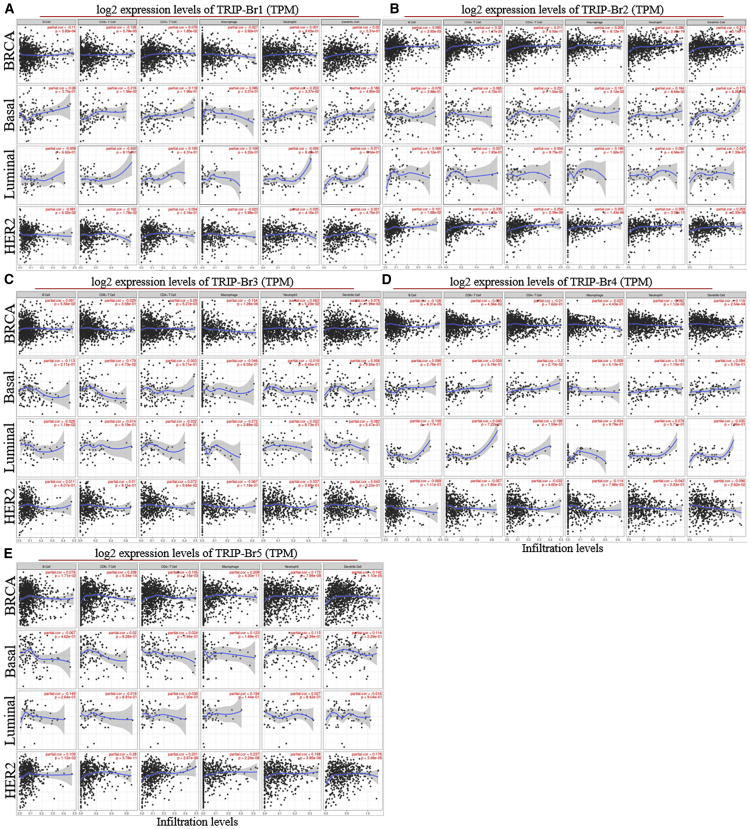
Correlations between TRIP-Br Expression and Immune Infiltrates in BRCA Patients (A–E) The level of TRIP-Brs was normalized and depicted with a log2 TPM (transcripts per million) value using TIMER RNA-seq datasets in BRCA patients samples.

To explore the correlation between expression of TRIP-Brs and the diverse immunological-infiltrating levels, we conducted the correlation analysis using the TIMER^36^ database. Interestingly, we found that overexpression of TRIP-Brs were linked with lower prognostic values by significant immune-infiltrating levels of CD4^+^ T cell (TRIP-Br-1/HR = 3.401; TRIP-Br-2/HR = 1.317; TRIP-Br-3/HR = 1.080; TRIP-Br-4/HR = 1.487; and TRIP-Br-5/HR = 1.191), macrophage (TRIP-Br-1/HR = 9.515; TRIP-Br-2/HR = 9.655; TRIP-Br-3/HR = 9.461; TRIP-Br-4/HR = 7.505; and TRIP-Br-5/HR = 7.781), and neutrophil (TRIP-Br-1/HR = 13.584; TRIP-Br-2/HR = 9.739; TRIP-Br-3/HR = 13.302; TRIP-Br-4/HR = 20.363; and TRIP-Br-5/HR = 15.116). In addition, we also observed the relation between TRIP-Brs and biomarkers of immune genes, including neutrophils, macrophages, CD4^+^ T cells, B cells, and CD8^+^ T cells, as well as dendritic cells (DCs), in BRCA (Figures 10A–10E). Indeed, TRIP-Br-1 was positively correlated with immune-infiltrating markers CD4^+^ T cells (Corr = 0.076, p = 1.85e–02), B cells, and DCs (Corr = 0.02, p = 5.31e–01) (Table S4). We also found a significant positive correlation of TRIP-Br-2 with macrophages (Corr = 0.205, p = 8.12e–11), CD8^+^ T cells (Corr = 0.32, p = 1.17e–24), CD4^+^ T cells (Corr = 0.211, p = 4.04e–11), and neutrophils (Corr = 0.286, p = 2.40e–19), as well as DCs (Corr = 0.211, p = 5.14e–11). TRIP-Br-3 was positively associated with only macrophages (Corr = 0.154, p = 1.28e–06). TRIP-Br-5 was positively correlated with macrophages (Corr = 0.208, p = 4.30e–11), CD4^+^ T cells (Corr = 0.105, p = 1.16e–03), CD8^+^ T cells (Corr = 0.238, p = 5.34e–14), and DCs (Corr = 0.142, p = 1.10e–05), as well as neutrophils (Corr = 0.173, p = 7.99e–08), whereas there was no significant correlation in TRIP-Br-4 with immune-infiltrating cell markers (Figure 10D). These results indicated that TRIP-Brs have a key function in the infiltration of immune cells in patients with BRCA.

Similarly, TRIP-Brs were involved in the infiltration of immunological markers in basal-, luminal-, and HER2-enriched BRCA patients. Interestingly, we found that TRIP-Brs were positively correlated with CD4^+^ T cells (except TRIP-Br-3/4, which were negatively correlated) in basal, luminal, and HER2 BRCA (Figures 10A–10E). On the same way, TRIP-Brs were positively correlated with neutrophil (except TRIP-Br-1/3/4, which were negatively correlated in luminal/basal/HER2) and DC (except TRIP-Br-3/4, which were negatively correlated in luminal/HER2) infiltration in basal-, luminal-, and HER2-enriched patients (Figures 10A–10E). TRIP-Brs were both positively and negatively associated with CD8^+^ T cells and macrophage infiltration in basal-, luminal-, and HER2-enriched BRCA; however, these factors were negatively correlated with B cell infiltration in all of the basal-, luminal-, and HER2-enriched patients, whereas TRIP-Br-1/4 (in basal), TRIP-Br-2 (in luminal/HER2), TRIP-Br-3/5 (in HER2) were positively correlated with B cell infiltration in patients with BRCA (Figures 10A–10E).

## Discussion

TRIP-Br nuclear factors appeared as principal regulators that are associated with the diverse cellular processes, including transcriptional control, cell cycle regulation, and various signaling cascades at transcriptional and translational levels.^6^, ^7^, ^8^, ^9^, ^10^, ^11^, ^12^ These factors participated in the initiation and development of many cancers, including BRCA. However, the potential functions of TRIP-Brs in the several cancers with prognostic values in patients have been partially elucidated; further investigation of the molecular correlation between TRIP-Brs and BRCA has yet to be investigated. In the TRIP-Br family, TRIP-Br-1 is reported as a potential factor in neuronal cell proliferation and BRCA.^11^^,^^17^ Some findings suggested that TRIP-Br-1 behaves like a transcriptional regulator, anti-apoptotic factor, cellular senescence regulator, and cell cycle modulator.^11^^,^^13^ Elevated levels of TRIP-Br-1 are associated with more invasion and more survival of BRCA cells via upregulation of tumorigenic CDK4, resulting in BRCA aggression and tumorigenesis.^9^^,^^32^, ^33^, ^34^, ^35^ In this study, we postulated the key functions of TRIP-Br-1 with various aspects, especially in BRCA. Interestingly, gene-rank analysis using Oncomine Cancer Genome Portal and TIMER online analysis showed that TRIP-Br-1 has secured the top rank and differentially overexpressed in multiple carcinomas, including human BRCA (Finak’s datasets, fold change = 3.77, p = 1.52E–27)^32^ samples, rather than respective normal tissues. Especially, TIMER^36^ analysis showed significant overexpression of TRIP-Br-1 in the breast (BRCA), bladder carcinoma (BLCA), CHOL, COAD, ESCA, head and neck squamous cell carcinoma (HNSC), kidney renal papillary cell carcinoma (KIRP), and LUSC with respect to normal tissue samples in TCGA datasets. Similarly, previous literature also reported that TRIP-Br-1 was overexpressed in nasopharyngeal cancer, cervical cancer, and melanoma and regulated by a chemotherapeutic drug and its synthesized decoy peptide.^48^ One finding worth mentioning revealed that TRIP-Br-1 halts the apoptosis process through stabilization of XIAP in BRCA.^11^ This phenomenon inspired us to elucidate the potential roles of TRIP-Br-1 in a subtype of BRCA development. In this study, TRIP-Br-1 was moderately expressed in most of the subtypes, including basal, luminal, and HER2-E positive of BRCA. To confirm further about the role of TRIP-Brs, we also conducted an extensive study with an *in vitro* blot, immunocytochemistry, and IHC of normal versus three types of BRCA—MCF7, MDA-MB-231, and BT-20—inoculated xenograft tumor model to comprehensively assess the expression of TRIP-Br protein in normal and BRCA cells. Interestingly, protein levels of TRIP-Br-1 were significantly higher in BRCA than the normal MCF10A cells. These results indicated the involvement of TRIP-Br-1 in BRCA carcinogenesis through several biological processes. Before analyzing the key functions of TRIP-Br-1 in diverse cellular mechanisms and pathways, firstly, we investigated the role of transcriptomic levels of TRIP-Br-1 on the prognostic value in patients with invasive BRCA. The significant higher mRNA levels of TRIP-Br-1 are linked with the clinical features in invasive BRCA patients. We next examined the prognostic values of TRIP-Br-1 using KM survival analysis in a BRCA patient cohort. An overexpression of TRIP-Br-1 was interestingly linked with the worse and poor OS, RFS, DMFS, and PFS in all of the BRCA patients in more than 150 months.

Another family member, TRIP-Br-2, has been well studied as a main regulator in multiple tumorigenesis processes through activation of E2F1/DP-1.^6^ However, potential roles of TRIP-Br-2 have not been investigated, especially in BRCA, to date. Earlier, Cheong et al.^22^ reported that TRIP-Br-2 is overexpressed in many tumor. Similarly, our findings showed higher levels of TRIP-Br-2 in several cancers, including BRCA, CHOL, LUSC, HNSC, KIRC, and ESCA, than normal tissues, and TRIP-Br-2 is moderately expressed in most BRCA subtypes. GEPIA analysis and *in vitro* results also revealed that TRIP-Br-2 was significantly overexpressed in both mRNA and protein levels in BRCA cells than normal MCF10A and tissue samples. The TRIP-Br-2 overexpression was affecting the number of the cellular programs not only in BRCA but also in other diseases. TRIP-Br-2 is involved in obesity, insulin resistance, and hyperlipidemia patients.^19^ The transcriptional changes in TRIP-Br-2 significantly regulate the functions of cyclin-E-induced cell division of cells.^7^ In addition, higher mRNA levels of TRIP-Br-2 are correlated with poor survival in hepatocellular carcinoma patients.^22^ In line with this finding, our results showed that a significant TRIP-Br-2 overexpression was linked with worse RFS, DMFS, OS, and PFS in BRCA patients. However, the KM survival using KM plotter showed that mRNA overexpression of TRIP-Br-2 was not associated with BRCA cancer stages in patients. Collectively, TRIP-Br-2 plays a significant role in BRCA induction with great clinicopathological and prognostic values.

It has been studied that TRIP-Br-3 significantly induced cancer progression in a nude mice model of TNBC.^49^ According to TIMER analysis, TRIP-Br-3 is found to be overexpressed in several cancers, including BRCA, BLCA, CHOL, ESCA, HNSC, liver hepatocellular carcinoma (LIHC), and LUSC, whereas significantly under or lower expressed in luminal/basal/HER2-positive BRCA and KICH than normal tissues. Another study also proposed the unique role of TRIP-Br-3: a short hairpin knockdown of TRIP-Br-3 significantly halted the propagation of BRCA cells up to 50%.^26^ These results indicate tumorigenic roles of TRIP-Br-3 in the proliferation of BRCA cells. Intriguingly, a deep analysis of the BRCA subtype showed that TRIP-Br-3 was highly expressed in basal-like and luminal subtypes of BRCA. The Oncomine analysis also revealed an overexpressed of TRIP-Br-3 in medullary breast carcinoma (fold = 2.302), mucinous BRCA (fold = 3.060), and invasive ductal breast carcinoma (fold = 2.25) as compared to normal adjacent patient tissues. Subsequently, we studied the influence of TRIP-Br-3, in particular, the tumor stage; it was significantly associated with stage II and stage III tumors in BRCA patients. In *in vitro* analysis, significant overexpression of TRIP-Br-3 in BT-20 and histopathological of MDA-MB-231 inoculated xenograft tumor samples. Similarly, Alderman and his team^50^ reported that TRIP-Br-3 significantly enhanced the tumor burden of melanoma cells in the xenograft model and repressed by tumor suppressor microRNA-15a. However, we did not find a significant difference at protein levels (low level) in histopathology of BRCA tissue with respect to normal tissue in the data from Protein Atlas (Figure 4C). TRIP-Br-3 was also overexpressed and involved in carcinogenesis of ovarian cancer with great prognostic accuracy.^51^ Similarly, overexpression of TRIP-Br-3 was linked with poor OS and PFS in patients with BRCA. Taken together, these data were depicting an involvement of TRIP-Br-3 in different types of cancers, including BRCA because it may be a fabulous oncogenic factor that modulates E2F-dependent activation at the transcriptomic level and regulates cell propagation.^23^ Therefore, it is with a dire need to study extensively about the mechanism action of TRIP-Br-3 in invasive BRCA.

Nuclear transcriptional modulator TRIP-Br-4^27^ plays carcinogenic functions, but the novel role of TRIP-Br-4, especially in BRCA, is unclear. One variant of TRIP-Br-4 modulates cell cycle phases at G1 and S via E2F1-associated transcriptional activities.^27^ An interesting study by Cho and his team^29^ deduced that overexpression of TRIP-Br-4 significantly controlled the cancer cell growth and enhanced cancer-transformed cells. In the same way, Oncomine, GEPIA, and TIMER analyses demonstrated that TRIP-Br-4 was highly carcinogenic and overexpressed in several cancers, such as BRCA, CHOL, HNSC, LIHG, and pancreatic cancer patients’ tissue with respect to normal tissues. In our *in vitro* analysis, we found that TRIP-Br-1 was expressed more highly than that normal cells in translational levels. It seems that mRNA and translational levels of TRIP-Br-4 participate in the governing of BRCA progression and metastasis. Therefore, we next investigated the expression status at different subtypes, stages, and grades of BRCA. Our results showed that TRIP-Br-4 was highly expressed in the luminal subtype of BRCA and impacted higher patient risk with tumor stage III (HR = 3.868) and stage IV (HR = 10.54), lymph node status (HR = 1.11), and tumor grade 1 (HR = 2.24), which define the TRIP-Br-4 oncogene that is upregulated in BRCA and enhanced tumorigenesis with poor prognosis. In addition, KM survival curve also demonstrated that TRIP-Br-4 significantly reduced OS, RFS, DMFS, and PFS after 200 months of BRCA patients’ survival.

The TRIP-Br-5 transcription factor has diverse roles in several biological processes,^6^, ^7^, ^8^ but distinct potential roles of TRIP-Br-5 have not been reported yet in BRCA cancer progression. Nuclear factor TRIP-Br-5 involves the cardiac fibroblast activation TGF-β-dependent manner.^30^ Kusano et al.^52^ revealed a significant role of TRIP-Br-5 in repressing transactivation of I-mfa through interaction with its SERTA domain by an E2F-1-mediated mechanism. E2F1 transcription factor functioned as a key regulator of cancer progression, including BRCA.^53^ However, the functional role of TRIP-Br-5, especially in BRCA, is not validated yet. In our present study, Oncomine gene rank showed TRIP-Br-5 significantly overexpressed in breast, kidney, lung, and pancreatic tumors than that normal breast samples. In the TIMER study, the mRNA levels of TRIP-Br-5 were highly expressed in BRCA, CHOL, ESCA, and LUSC, whereas significant downregulation was found in only thyroid cancer (THCA) and rectum adenocarcinoma (READ), according to TCGA datasets (Figure 1F). Then, we further investigated the mRNA levels of TRIP-Br-5 in the BRCA subtype and found higher expression in basal-like BRCA. However, it was not significantly expressed in other subtypes of BRCA. Further, Oncomine database analysis showed that TRIP-Br-5 was significantly associated with invasive ductal breast carcinoma (fold = 2.151), tubular breast carcinoma (fold = 1.666), and invasive ductal breast carcinoma stroma (fold = 1.812) (Table 1) as compared to normal tissues. Simultaneously, GEPIA analysis also indicated an elevated mRNA fold of TRIP-Br-5 (dot and box-whisker plot) in BRCA than that of normal breast tissues. Similarly, we also examined the expression levels of the TRIP-Br-5 oncoprotein in normal versus BRCA cell lines and in human tissues. Histopathological analysis of xenograft tumors has also shown moderate to higher expression of TRIP-Br-5 in MCF7 and BT-20 inoculated tumor tissues. On the same way, Protein Atlas database revealed a medium IHC staining of TRIP-Br-5 in human BRCA patients’ samples than low staining in normal breast tissues. These *in silico* and *in vitro* analyses defined that the higher mRNA or protein levels of TRIP-Br-5 are linked with poor prognostic values (reduced OS) in BRCA patients. On the same way, cBioPortal BRCA dataset analysis also depicted that alteration and expression of TRIP-Br-5 resulted in worse OS, PFS, and DFS (Figure 3). Univariate analysis revealed that age at diagnosis, grades, and stages, together with TRIP-Br-5, were statistically significant factors for BRCA progression.

After elucidation, the impact of TRIP-Br expression, and prognosis in patients with BRCA, we also studied the association between TRIP-Br expression and risk of BRCA patients analyzed by Cox proportional hazard regression model with OS, PFS, and RFS. An overexpression of tumorigenic TRIP-Br-2 (HR = 1.24, I^2^ = 0%, τ^2^ = 0.0, p = 0.87) was observed in BRCA patients and significantly associated with high risk and worse PFS. It was noted that increased TRIP-Br-3 (PFS, HR = 1.28, I^2^ = 18%, τ^2^ = 0.0563, p = 0.29) was linked with worse PFS, whereas enhanced TRIP-Br-5 (PFS, HR = 0.94, I^2^ = 23%, τ^2^ = 0.0098, p = 0.18) significantly associated with better prognosis in all BRCA patients (Figure 7). This study indicated the over mRNA levels of TRIP-Brs were associated with significant prognosis with OS, PFS, and RFS of BRCA patients. So, we hypothesized that TRIP-Brs may play a significant role in BRCA initiation, recurrence, and metastasis that need further investigation to elaborate. Genetic mutation or alteration in the particular gene also influences the prognosis of patients with BRCA.^54^ Here, we found that nuclear factor TRIP-Brs were mutated in 1,156 of queried samples (17%) of 5,700 total patients and significantly reduced the patients’ OS (p = 0.189), DFS (p = 0.0138), PFS (p = 6.522e–3), and DSFS (p = 0.0254) in the altered group as compared with unaltered patients. In contrast, different types of mutation for each TRIP-Br were also observed in mutated BRCA, as well as various other cancer samples. These factors are together involved and correlated with each other to enhance tumor progression in BRCA. Similarly, TRIP-Brs associated E2F transcriptions correlated with each other and control BRCA.^53^ In our analysis, Pearson’s correction was defined as significant positive as well as negative correlation in TRIP-Brs with each other, along with associated genes in RNA-seq GEPIA and TCSBN BRCA datasets, as compared to normal breast mammary tissues. In addition, some genes were also correlated with expression and clinicopathological significance of TRIP-Brs in patients with BRCA. Therefore, it is worth to find more about the mechanism actions of mutated TRIP-Brs along with associated genes in BRCA tumor patients to exploit potential signaling cascades, biological processes, as well as molecular functions. Further, the data were subjected to analyze GO pathways in mutated TRIP-Brs with associated genes through the KEGG. In this study, cBioPortal analysis showed that multiple mutations in the TRIP-Br family significantly regulate the number of mutated genes in BRCA patients and induce BRCA through several biological mechanisms. Differentially mutated genes, along with TRIP-Brs, were involved in the regulation of gene expression, macromolecule biosynthetic mechanisms, nucleic acid-transcriptional steps, cardiovascular activities, single-organism formation, steroid hormone receptor, leukocyte cell-cell adhesion function, and cell cycle process, as reported previously.^55^ GO_CC revealed that intracellular organelle lumen, macromolecular complex, Z disc, myofibril, nucleoplasm, I band, cell projection, costamere, dendrite, SWI/SNF complex, as well as neuron spine were also significantly controlled by mutation of TRIP-Br in patients with BRCA. The binding process of protein transcription factor, calcium ion, actin filament binding and nucleic DNA, ATP, and androgen receptor was also regulated by a mutation in TRIP-Brs and associated factors in BRCA. KEGG pathways’ investigation confirmed the pathways associated with the role of mutations in TRIP-Brs and other mutated neighbor genes; thyroid hormone signaling pathway, ErbB signaling pathway, small cell lung cancer, hepatitis B, endometrial cancer, PI3K-Akt signaling pathway, chronic myeloid leukemia, central carbon metabolism in cancer, cAMP signaling pathway, cell cycle, glioma, neurotrophin cellular pathway, and calcium channel pathways were significantly linked with the mutational changes in TRIP-Brs in BRCA patients. Apart from these, the potential roles of TRIP-Brs in BRCA and other cellular processes through several biological pathways, including E2F1 proliferation pathway, neurological process, PI3K/AKT/BRCA1 axis signaling pathway,^56^ adenylyl cyclase-mediated cAMP signaling,^12^ Nrf pathway,^26^ ER stress-induced BAT dysfunction,^57^^,^^58^ BMP/SMAD pathway,^58^ reactive oxygen species (ROS)-induced cell death and ubiquitination,^11^^,^^15^ p53 transcriptional and pRb1-cyclin D1-cdk4/6-p16(INK4A) pathway,^59^ Toll-like receptor (TLR), induced immune.^18^^,^^27^^,^^60^, ^61^, ^62^ Further, this study also revealed the correlation between mRNA levels of TRIP-Brs and immune-infiltrating factors. Thus, TRIP-Brs may also play an essential role in spatial heterogeneity of the tumor microenvironment in patients with BRCA. Presently, very little is deciphered about the potential roles of TRIP-Brs in the regulation of the immunological responses. It has been reported that TRIP-Brs were associated in regulating immune-infiltrating cells, such as macrophages, CD4^+^ T cells, CD8^+^ T cells, and DCs, as well as neutrophil cells in patients with BRCA. One similar study also indicated that onco-factor TDO2 regulates an immune infiltrate in BRCA.^63^ However, a detailed study between TRIP-Brs and more immunological factors remains to be elucidated. Therefore, further extensive studies to determine the potential roles of TRIP-Brs in the modulation of immunological responses might propose a new paradigm for TRIP-Br-mediated immunotherapeutic strategies.

In the present study, we comprehensively studied the levels of all TRIP-Brs at transcriptomic and translational levels in BRCA. We also proposed a novel paradigm to elucidate the complexity and heterogeneity of BRCA with TRIP-Brs. This study suggested that overexpression of TRIP-Br-1 (fold change 3.771; t test = 20.664), -2 (invasive BRCA with mRNA levels 1.380, lobular BRCA 1.164, and ductal BRCA 1.025), -3 (medullary breast carcinoma with 2.302 mRNA fold change), and -5 (invasive ductal breast carcinoma stroma, Ma breast4 statistics, fold change = 1.812) in breast samples may be critical for the initiation of BRCA. Elevated levels of TRIP-Br-1, -2, -4, and -5 could also contribute as specific indicators to recognize poor prognosis in BRCA. Our investigations revealed that TRIP-Br-1, -2, and -5 act as molecular targets in patients with BRCA, and transcription factor TRIP-Br-3 and TRIP-Br-4 were also observed as key prognosis factors for the management of BRCA with significant prognostic values.

## Materials and Methods

### Oncomine Database Analysis

A comprehensive comparison between BRCA patient specimen and adjacent normal tissue specimen datasets was investigated using the Oncomine^31^ gene-expression microarray database system. The Oncomine contains about 715 total datasets with 86,733 samples of several cancers that enhance readability research based on a genome-wide expression profile. The mRNA expression and clinicopathological significance of TRIP-Brs were comprehensively analyzed with a threshold set: top 10% gene ranking, fold change of 2, p <0.05, and gene expression was measured as mRNA levels. The transcriptional fold of each TRIP-Br in various types of human carcinoma was investigated in this Oncomine online system.

### GEPIA Dataset Analysis

TRIP-Br expression, subtype analysis, differential expression, correlation, and stage plots were performed using GEPIA.^37^ The GEPIA database encloses GTEx and TCGA that contain 9,736 cancer and 8,587 normal tissue specimens in patients with BRCA. It distinctly delivers customizable functions in tumor versus normal samples through differential expression analysis, gene profiling based on carcinoma and stages, patients’ prognosis, genes correlation, and dimensionality reduction analysis.

### TRIP-Br Expression Analysis via *In Vitro* and *In Vivo* Study

Three types of BRCA cell lines: luminal subtype MCF7 (ER^+^, PR^+^, and human HER2^−^), basal B subtype MDA-MB-231, and basal A BT-20 (ER^−^, PR^−^, and HER2^−^) cells were propagated in DMEM high glucose medium and RPMI-1640 media containing 1% antibiotics and 10% fetal bovine serum (FBS) and at a steady-state conditioned CO_2_ incubator, as reported previously.^64^

The induction of the xenograft for the elucidation of expression of TRIP-Brs in different BRCA proposals was approved by the reviewing academic committee of the Animal Facility Center, Sookmyung Women’s University (Seoul, Republic of Korea). All of the protocol for xenograft induction was performed according to the principles communicated in the Declaration of Helsinki. Briefly, MCF7, BT-20, and MDA-MB-231 cells were harvested and 1 × 10^6^ cells/100 μL inoculated onto the lower-right and left flank of nude mice. The mice were sacrificed and subjected to perform immunohistopathological analysis.

### Immunohistopathological Analysis

IHC of xenograft tissues, 5 μm-thick tumor sections were stained with commercially available anti-mouse and anti-rabbit antibodies for TRIP-Br-1 (mouse; Enzo Life Sciences), TRIP-Br-2 (Abcam; ab272581), TRIP-Br-3 (rabbit; ab227969), TRIP-Br-4 (ab107728), and TRIP-Br-5 (rabbit; polyclonal LS Bio-LS-C325020) at a 1:200 concentration overnight at 4°C. The next day, the slides were counterstained with horseradish peroxidase (HRP)-conjugated secondary antibodies (1:500 concentration; Santa Cruz Biotechnology) at room temperature (RT) for 3 h. After that, tissue-fixed glass slides were exposed with 3,3′-diaminobenzidine (DAB) substrate (Vector Laboratories). After, mounting of slides was kept for 1 h at RT and then observed under light microscopy (Olympus, Tokyo, Japan).

### Genetic Mutation, Expression, and Clinicopathological Analysis by cBioPortal

The cBioPortal is an interactive multidimensional web server that is associated with the Memorial Sloan Kettering Cancer Center. The cBioPortal^46^ is an open interactive web server (http://www.cbioportal.org/) that enables visualization at large-scale cancer genome datasets. In addition, cBioPortal contains about 105 studies with more than 4,000 patients’ samples of different cancers in TCGA pipeline. Hence, we investigated the potential roles of TRIP-Brs in patients with BRCA, especially in terms of genetic mutations (missense mutation, deep deletion, amplification, copy number variance) and prognostic values of patients with BRCA. In addition, GISTC and *Z* scores of mRNA expression for RNA-seq datasets were surfed by the cBioPortal system.

### Functional Enrichment, Annotation, GO, and Pathway Analysis

TCGA and BRCA datasets were retrieved from cBioPortal, and selected top 100 mutated genes that associated with a genetic aberration in TRIP-Brs in patients with BRCA were subjected to perform gene-enrichment analysis. The GO_BP, GO_CC, and GO_MF, as well as associated KEGG signaling pathways, were analyzed using the ggplot2 package^65^ in statistical software R, v.i386 4.0.0. Further, the significant GOs and the KEGG pathways were analyzed. The significant p value (p < 0.05) was opted in all the tests. In addition, we also analyzed gene-coexpressed, top-associated genes and GO, as well as pathways using Metascape^66^ (http://metascape.org) and Coexpedia^67^ analysis tool for gene annotation and analysis.

### Patients’ Survival and Hazardous Risk Analysis

In this study, mRNA levels of TRIP-Brs and clinicopathological significance were studied using the survival KM Plotter (https://kmplot.com/analysis/).^44^^,^^45^ KM Plotter contains BRCA gene-expression datasets (n = 6,234). The gene expression versus survival of patients with BRCA was generated mainly from three data sources, like cancer Biomedical Informatics Grid, TCGA, and GEO. Contrast, patients OS, RFS, DMFS, and PFS with BRCA were measured based on median expression value and 95% CI, and log rank test p value <0.05 was assumed as statistically significant. In addition, we also investigated the survival and meta-survival analysis for patients’ hazardous risk using the GENT2 database system,^68^ and significant RFS for TRIP-Br-1 was analyzed using the PrognoScan^69^ server.

### Expression, Subtype, Tumor Stages, and Clinical Features Analysis

bc-GenExMiner v.4.4^41^ is an online microarray interactive platform of investigated datasets of BRCA. It contains (DNA microarrays [n = 10,001] and RNA-seq [n = 4,712]) samples of BRCA. Targeted expression in various datasets, combined TCGA DNA-seq, RNA-seq, and GEO datasets were determined using the targeted correlation, targeted expression, and prognostic modules. In the analysis, we considered statistically p value by Welch’s test. In addition, we also elucidated the expression of TRIP-Brs in more than two groups for significant difference in pairwise analysis (p < 0.05) using the Dunnett-Tukey-Kramer’s test. Lastly, we also investigated the meta-survival analysis for patients’ hazardous risk using the GENT2 database system.^67^

### Independent RNA-Seq Data Analysis Using GOBO Database Analysis

The TRIP-Br expression was investigated using GOBO database in ER^+/−^- and PR^+/−^-enriched subtypes, along with a different tumor grade of BRCA. GOBO is a web server database that contains 1,881 samples of BRCA patients.^40^

### TRIP-Br-Associated Immune-Infiltrating Analysis

The TIMER^36^ is an online platform that enables investigation of the correlation, expression, and prognosis of particular tumor immune-infiltrating immunological responses. TIMER has large-scale datasets that contain about 10,897 specimens from 32 cancer patients, including BRCA. Module diffExp was used to elucidate the differential expression of TRIP-Brs in multiple cancers based on a Wilcoxon statistical test. The association between mRNA levels of TRIP-Brs and tumor-infiltrating cells (especially macrophages, B cells, neutrophils, CD8 T cells, CD4 T cells, and DCs) was determined using the module gene. The Spearman correlation analysis performed to investigate the expression of TRIP-Brs and associated immune infiltrate in BRCA samples that was depicted by scatterplots. The correlation module was followed to correlate immune marker gene sets, and TRIP-Br expression followed earlier studies.

### Statistical Analysis

The survival analysis of BRCA patients was performed using the KM Plotter web tool, and set significance threshold followed default settings. The significant p value was calculated using log rank test p value 0.05. In the Oncomine study, t tests were conducted both as two sided for differential expression and one sided for specific overexpression analysis. The heterogeneity was tested using the I^2^ index and Cochran’s Q test, with significant heterogeneity assumed for I^2^ >50% or a Q test p value <0.05. Statistical significance of the data (p values) was provided by the program. The Spearman correlation coefficient statistical comparison is used to determine correlation.

## Author Contributions

Conceptualization, R.K.M., C.B.M., and M.-S.L.; Methodology, R.K.M., C.B.M., B.S.L., and S.J.; Funding Acquisition, M.-S.L. and S.J.; Investigation, R.K.M. and C.B.M.; Project Administration, S.J., B.S.L., and M.-S.L.; Supervision, M.-S.L.; Visualization, R.K.M., S.J., N.T.N.Q., N.H.A., D.M., and T.J.; Writing – Original Draft, R.K.M. and C.B.M.; Formal Analysis, R.K.M.; Writing – Review & Editing, R.K.M., C.B.M., and M.-S.L.

## Conflicts of Interest

The authors declare no competing interests.

## Supplemental Information

Document S1. Figures S1–S6 and Tables S1–S7

Document S2. Article plus Supplemental Information
