# Supplementary material for: Exploring the Role of TRIP-Brs in Human Breast Cancer: An Investigation of Expression, Clinicopathological Significance, and Prognosis
Source: Mol Ther Oncolytics. 2020 Sep 16;19:105–26. doi: 10.1016/j.omto.2020.09.003 (PMC7554327; doi:10.1016/j.omto.2020.09.003)
Supplement: Document S1. Figures S1–S6 and Tables S1–S7 [file mmc1.pdf]

OMTO, Volume *19*

Supplemental Information

Exploring the Role of TRIP-Brs in Human Breast Cancer: An Investigation of Expression, Clinicopathological Significance, and Prognosis

Raj Kumar Mongre, Chandra Bhushan Mishra, Samil Jung, Beom Suk Lee, Nguyen Thi Ngoc Quynh, Nguyen Hai Anh, Davaajragal Myagmarjav, Taeyeon Jo, and Myeong-Sok Lee

Supplementary Information

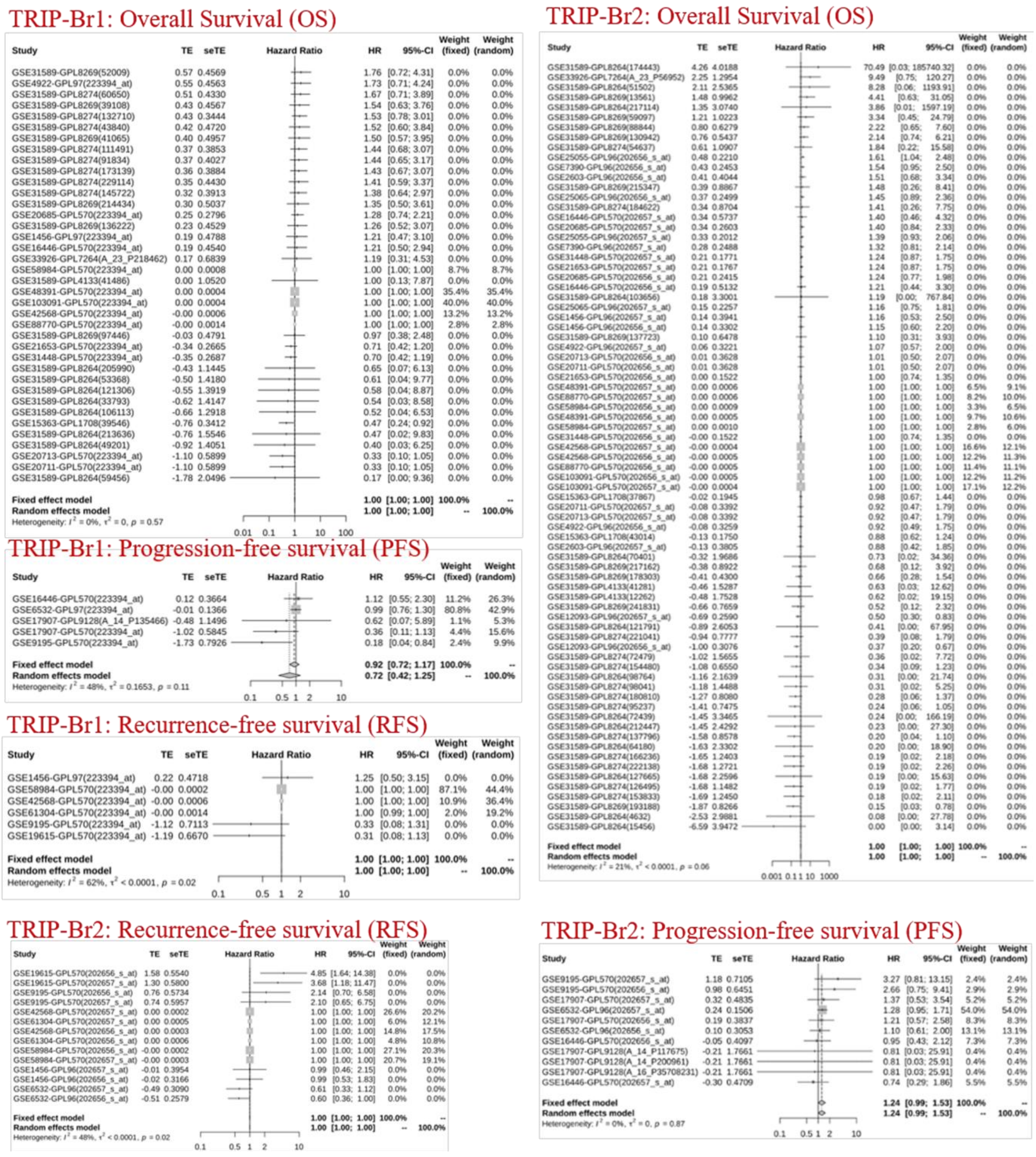

Figure S1. Cox log rank test regression analysis of TRIP-Brs expression and BRCA patient's survival. Hazard rate as BRCA patients cancer risk for OS, PFS, and RFS based on Fixed and Random effect model. It was elucidated that correlation between mRNA levels of TRIP-Br1/2 and affected hazard rate in BRCA patients. Abbreviations: PFS, progression-free survival; OS, overall survival; RFS, recurrence-free survival.

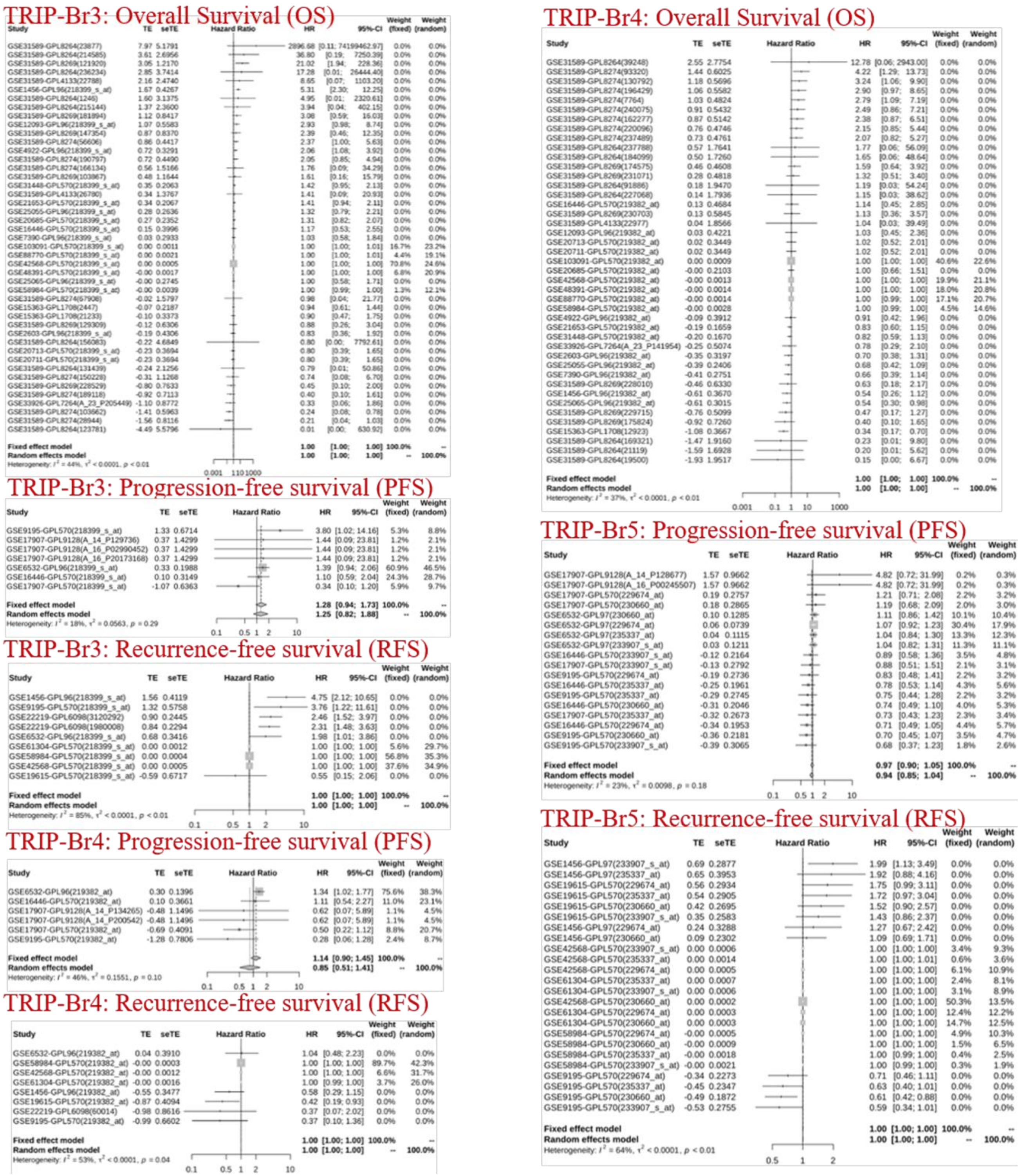

Figure S2. **Cox log rank test regression analysis of TRIP-Brs expression and BRCA patient's survival.** Hazard rate as BRCA patients cancer risk for OS, PFS, and RFS based on Fixed and Random effect model. It was elucidated that correlation between mRNA levels of TRIP-Br3/4/5 and affected hazard rate in BRCA patients. Abbreviations: PFS, progression-free survival; OS, overall survival; RFS, recurrence-free survival.

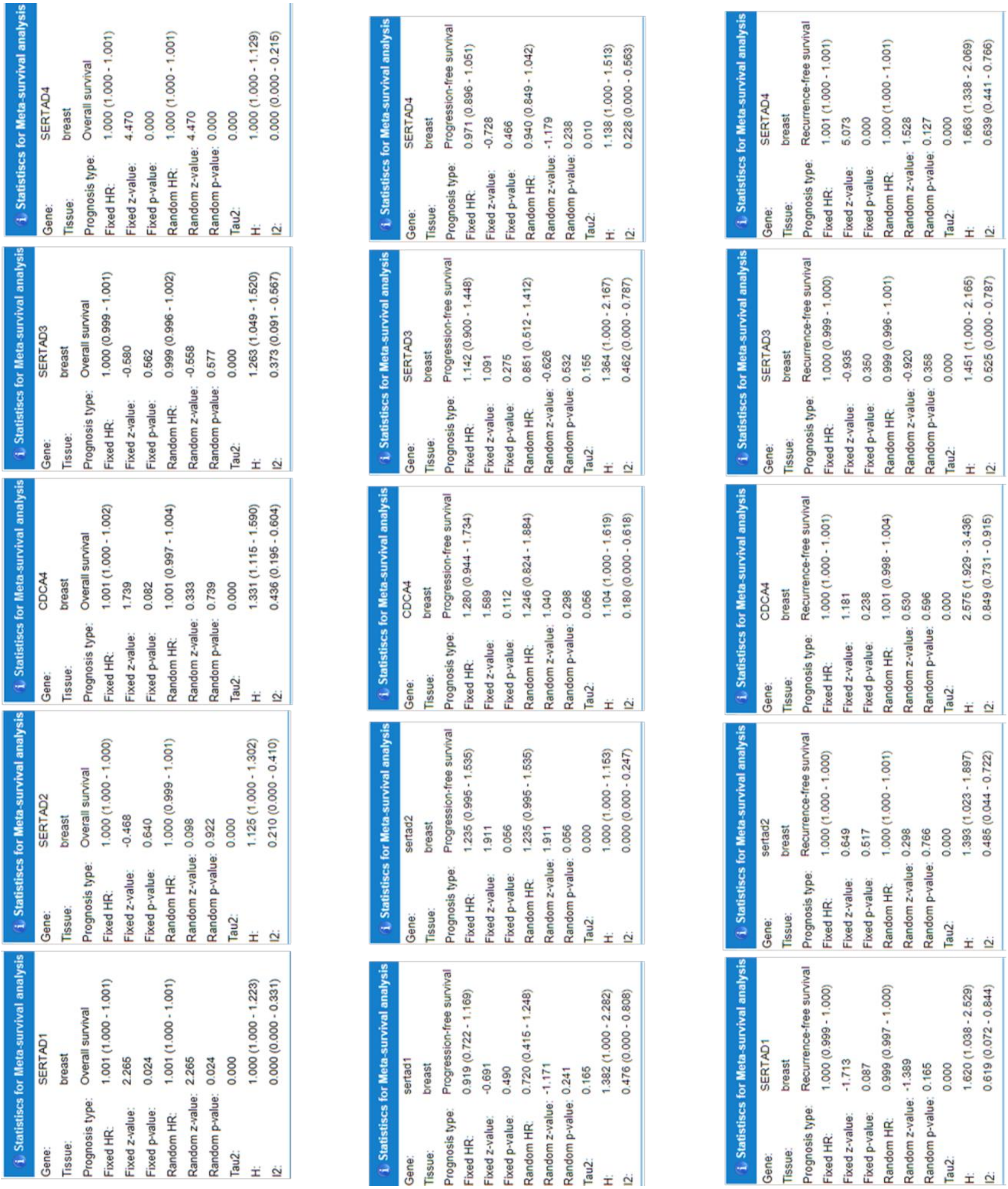
Statistiscs for Meta-survival analysis

Gene:	SERTAD1	SERTAD2	CDCA4	SERTAD3	SERTAD4
Tissue:	breast	breast	breast	breast	breast
Prognosis type:	Overall survival	Overall survival	Overall survival	Overall survival	Overall survival
Fixed HR:	1.001 (1.000 - 1.001)	1.000 (1.000 - 1.000)	1.001 (1.000 - 1.002)	1.000 (0.999 - 1.001)	1.000 (1.000 - 1.001)
Fixed z-value:	2.265	-0.468	1.739	-0.580	4.470
Fixed p-value:	0.024	0.640	0.082	0.562	0.000
Random HR:	1.001 (1.000 - 1.001)	1.000 (0.999 - 1.001)	1.001 (0.997 - 1.004)	0.999 (0.996 - 1.002)	1.000 (1.000 - 1.001)
Random z-value:	2.265	0.098	0.333	-0.558	4.470
Random p-value:	0.024	0.922	0.739	0.577	0.000
Tau2:	0.000	0.000	0.000	0.000	0.000
H:	1.000 (1.000 - 1.223)	1.125 (1.000 - 1.302)	1.331 (1.115 - 1.590)	1.263 (1.049 - 1.520)	1.000 (1.000 - 1.129)
I2:	0.000 (0.000 - 0.331)	0.210 (0.000 - 0.410)	0.436 (0.195 - 0.604)	0.373 (0.091 - 0.567)	0.000 (0.000 - 0.215)

Gene:	sertad1	sertad2	CDCA4	SERTAD3	SERTAD4
Tissue:	breast	breast	breast	breast	breast
Prognosis type:	Progression-free survival	Progression-free survival	Progression-free survival	Progression-free survival	Progression-free survival
Fixed HR:	0.919 (0.722 - 1.169)	1.235 (0.995 - 1.535)	1.280 (0.944 - 1.734)	1.142 (0.900 - 1.448)	0.971 (0.896 - 1.051)
Fixed z-value:	-0.691	1.911	1.589	1.091	-0.728
Fixed p-value:	0.490	0.056	0.112	0.275	0.466
Random HR:	0.720 (0.415 - 1.248)	1.235 (0.995 - 1.535)	1.246 (0.824 - 1.884)	0.851 (0.512 - 1.412)	0.940 (0.849 - 1.042)
Random z-value:	-1.171	1.911	1.040	-0.626	-1.179
Random p-value:	0.241	0.056	0.298	0.532	0.238
Tau2:	0.165	0.000	0.056	0.155	0.010
H:	1.382 (1.000 - 2.282)	1.000 (1.000 - 1.153)	1.104 (1.000 - 1.619)	1.364 (1.000 - 2.167)	1.138 (1.000 - 1.513)
I2:	0.476 (0.000 - 0.808)	0.000 (0.000 - 0.247)	0.180 (0.000 - 0.618)	0.462 (0.000 - 0.787)	0.228 (0.000 - 0.563)

Gene:	SERTAD1	sertad2	CDCA4	SERTAD3	SERTAD4
Tissue:	breast	breast	breast	breast	breast
Prognosis type:	Recurrence-free survival	Recurrence-free survival	Recurrence-free survival	Recurrence-free survival	Recurrence-free survival
Fixed HR:	1.000 (0.999 - 1.000)	1.000 (1.000 - 1.000)	1.000 (1.000 - 1.001)	1.000 (0.999 - 1.000)	1.001 (1.000 - 1.001)
Fixed z-value:	-1.713	0.649	1.181	-0.935	5.073
Fixed p-value:	0.087	0.517	0.238	0.350	0.000
Random HR:	0.999 (0.997 - 1.000)	1.000 (1.000 - 1.001)	1.001 (0.998 - 1.004)	0.999 (0.996 - 1.001)	1.000 (1.000 - 1.001)
Random z-value:	-1.389	0.298	0.530	-0.920	1.528
Random p-value:	0.165	0.766	0.596	0.358	0.127
Tau2:	0.000	0.000	0.000	0.000	0.000
H:	1.620 (1.038 - 2.529)	1.393 (1.023 - 1.897)	2.575 (1.929 - 3.436)	1.451 (1.000 - 2.165)	1.663 (1.338 - 2.069)
I2:	0.619 (0.072 - 0.844)	0.485 (0.044 - 0.722)	0.849 (0.731 - 0.915)	0.525 (0.000 - 0.787)	0.639 (0.441 - 0.766)

Figure S3. Summary of Cox log rank test regression analysis of TRIP-Brs expression and BRCA patient's survival.

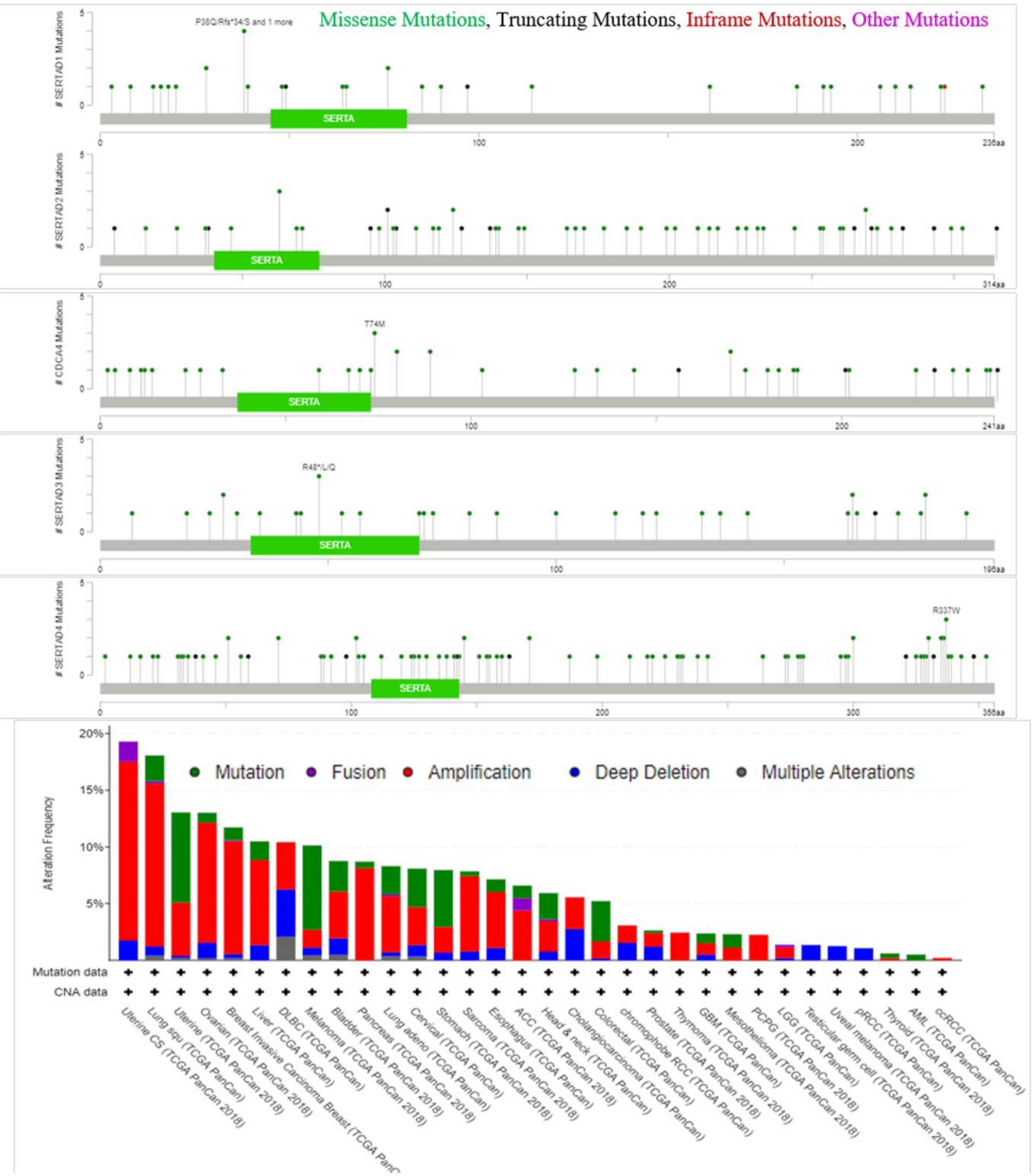

Figure S4. Presentation of mutational hotspots lollypop graphs of TRIP-Brs expression in BRCA patients.

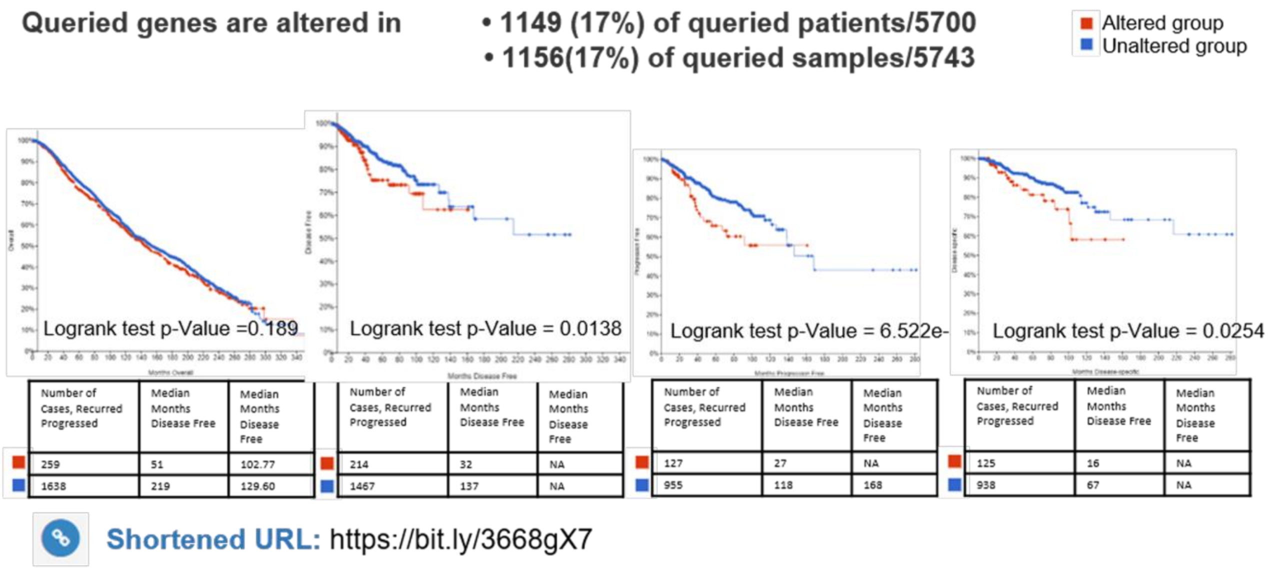

Figure S5. Genomic alterations in TRIP-Brs were significantly control survival of patients with BRCA.

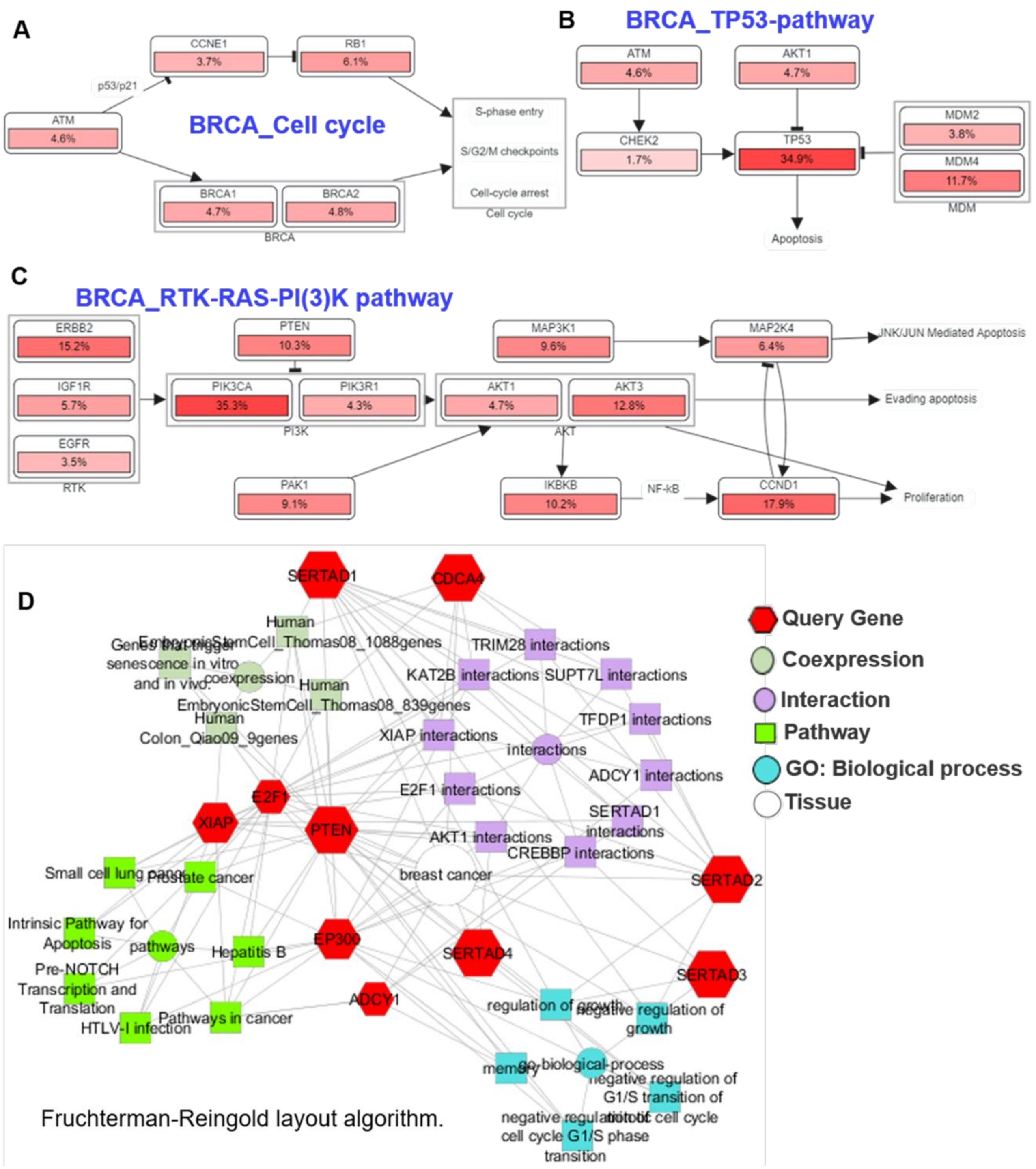

Figure S6. The Most Associated Pathways, Diverse Processes of Gene Coexpression, and Interacted Onco-factors in Breast Cancer. (A-C) cBioPortal analysis showed frequently altered genes were associated with mutation of TRIP-Brs in Cell cycle, TP53 pathway and RTK_RAS_PI(3)K pathway in BRCA patients. (D) Investigated queried genes are presenting significant GO, coexpression and interaction for breast cancer and Fruchterman-Reingold Layout Algorithm analysis showed an abstracted network for coexpression, interaction and pathways in BRCA tissues.

Table S1. An elevated levels of TRIP-Brs expression associated with clinical pathological characteristics (logistic regression) in patients with BRCA: Cox Proportional Hazard Model

Clinicopathologic variable	HR (95% CI_l-95% CI_U)	p-Value	Significant
**SERTAD1 or TRIP-Br1**			
Age (continuous)	1.037 (1.022-1.053)	0.000	***
Gender (Female vs. Male)	1.258 (0.173-9.157)	0.821	
Stage2(continuous)	1.605 (0.841-3.063)	0.152	
Stage3 (continuous)	3.893 (2.006-7.554)	0.000	***
Stage4 (continuous)	13.741 (6.067-31.125)	0.000	***
Grade 1	0 (0-inf)	0.0382	*
Grade 2	0.84 (0.27-2.65)	0.7628	
Grade 3	0.96 (0.58-1.59)	0.868	
Lymph node (Positive vs. Negative)	0.81 (0.48-1.38)	0.4469	
Purity	1.304 (0.444-3.827)	0.629	
B Cell	0.243 (0.002-29.211)	0.563	
CD8+ T Cell	0.217 (0.016-2.900)	0.248	
CD4+ T Cell	3.401 (0.057-201.934)	0.557	
Macrophage	9.515 (0.524-172.832)	0.128	
Neutrophil	13.584 (0.041-4512.451)	0.378	
Dendritic Cell	0.535 (0.062-4.593)	0.568	
SERTAD1 expression (low vs. high)	0.670 (0.496-0.904)	0.009	**
Wald test p =		3.78e-11	
Score (logrank) test		2.41e-18	
**SERTAD2 or TRIP-Br2**			
Age (continuous)	1.037 (1.021-1.052)	0.000	***
Gender (Female vs. Male)	1.096 (0.151-7.961)	0.928	
Stage2(continuous)	1.825 (0.963-3.456)	0.065	
Stage3 (continuous)	4.050 (2.094-7.831)	0.000	***
Stage4 (continuous)	11.437 (5.231-25.006)	0.000	***
Grade 1	0.98 (0.4-2.37)	0.9555	
Grade 2	1.42 (0.93-2.19)	0.1066	
Grade 3	1.36 (0.98-1.88)	0.0674	
Lymph node (Positive vs. Negative)	0.89 (0.6-1.31)	0.5421	
Purity	1.143 (0.417-3.131)	0.795	
B Cell	0.471 (0.005-44.930)	0.746	
CD8+ T Cell	0.222 (0.015-3.252)	0.272	
CD4+ T Cell	1.317 (0.025-69.633)	0.892	
Macrophage	9.655 (0.550-169.433)	0.121	
Neutrophil	9.739 (0.033-2859.237)	0.432	
Dendritic Cell	0.475 (0.055-4.081)	0.498	
SERTAD2 expression (low vs. high)	1.227 (0.949-1.587)	0.119	
Wald test p =		3.46e-12	
Score (logrank) test		1.51e-16	
**CDCA4 or TRIP-Br3**			
Age (continuous)	1.035 (1.020-1.051)	0.000	***
Gender (Female vs. Male)	1.126 (0.155-8.181)	0.906	
Stage2(continuous)	1.768 (0.934-3.345)	0.080	
Stage3 (continuous)	3.888 (2.017-7.494)	0.000	***
Stage4 (continuous)	11.106 (5.090-24.236)	0.000	***
Grade 1	2.09 (0.73-5.94)	0.1589	
Grade 2	1.09 (0.71-1.68)	0.6866	

Grade 3	1.13 (0.81-1.56)	0.4697	
Lymph node (Positive vs. Negative)	1.07 (0.73-1.58)	0.7271	
Purity	1.207 (0.438-3.327)	0.716	
B Cell	0.497 (0.005-45.724)	0.762	
CD8+ T Cell	0.351 (0.025-4.921)	0.437	
CD4+ T Cell	1.080 (0.022-53.705)	0.969	
Macrophage	9.461 (0.552-162.264)	0.121	
Neutrophil	13.302 (0.047-3740.619)	0.368	
Dendritic Cell	0.485 (0.057-4.113)	0.507	
CDCA4 expression (low vs. high)	1.080 (0.803-1.451)	0.611	
Wald test p		4.03e-12	
Score (logrank) test		2.36e-16	
**SERTAD3 or TRIP-Br4**			
Age (continuous)	1.036 (1.021-1.051)	0.000	***
Gender (Female vs. Male)	1.217 (0.168-8.841)	0.846	
Stage2(continuous)	1.707 (0.900-3.235)	0.101	
Stage3 (continuous)	3.868 (2.008-7.450)	0.000	***
Stage4 (continuous)	10.548 (4.826-23.057)	0.000	***
Grade 1	2.24 (0.88-5.75)	0.0847	
Grade 2	1.11 (0.72-1.7)	0.6441	
Grade 3	1.01 (0.73-1.4)	0.9414	
Lymph node (Positive vs. Negative)	1.11 (0.76-1.64)	0.5835	
Purity	1.183 (0.431-3.243)	0.745	
B Cell	0.317 (0.004-27.959)	0.615	
CD8+ T Cell	0.261 (0.020-3.450)	0.308	
CD4+ T Cell	1.487 (0.031-70.592)	0.840	
Macrophage	7.505 (0.461-122.236)	0.157	
Neutrophil	20.363 (0.073-5641.670)	0.294	
Dendritic Cell	0.427 (0.051-3.587)	0.433	
SERTAD3 expression (low vs. high)	0.710 (0.537-0.939)	0.016	*
Wald test p		2.01e-13	
Score (logrank) test		9.24e-18	
**SERTAD4 or TRIP-Br5**			
Age (continuous)	1.036 (1.022-1.050)	0.000	***
Gender (Female vs. Male)	0.590 (0.081-4.287)	0.602	
Stage2(continuous)	1.866 (0.038-3.357)	0.037	*
Stage3 (continuous)	3.774 (2.037-6.993)	0.000	***
Stage4 (continuous)	11.491 (5.426-24.338)	0.000	***
Grade 1	0.35 (0.03-4.03)	0.3789	
Grade 2	2.47 (0.74-8.22)	0.127	
Grade 3	1.73 (1.03-2.9)	0.0365	*
Lymph node (Positive vs. Negative)	1.87 (1.08-3.22)	0.0222	*
Purity	1.135 (0.410-3.144)	0.807	
B Cell	0.438 (0.005-41.560)	0.722	
CD8+ T Cell	0.256 (0.018-3.734)	0.319	
CD4+ T Cell	1.191 (0.023-61.578)	0.931	
Macrophage	7.781 (0.447-135.464)	0.159	
Neutrophil	15.116 (0.052-4425.084)	0.349	
Dendritic Cell	0.474 (0.055-4.099)	0.498	
SERTAD4 expression (low vs. high)	1.084 (0.932-1.260)	0.295	
Wald test p		5.2e-12	
Score (logrank) test		2.48e-16	

Table S2. Top correlated genes and association (Edge) with TRIP-Brs in BRCA patient's tissues

Source	Target	Score	P-value
SERTAD1	JOSD2	0.615178	6.30E-113
SERTAD1	RBM42	0.592146	1.03E-102
SERTAD1	LAMTOR4	0.575795	6.00E-96
SERTAD1	SDHAF1	0.574661	1.71E-95
SERTAD1	RABAC1	0.573493	5.02E-95
SERTAD1	MYL6	0.569543	1.84E-93
SERTAD1	NOSIP	0.56496	1.14E-91
SERTAD1	INAFM1	0.563779	3.25E-91
SERTAD1	FIS1	0.561679	2.09E-90
SERTAD1	ZNF524	0.551856	1.05E-86
SERTAD1	SELENOW	0.551729	1.17E-86
SERTAD1	DAPK3	0.551522	1.40E-86
SERTAD1	EIF3K	0.551429	1.51E-86
SERTAD2	PAPOLG	0.549744	6.33E-86
SERTAD2	STRN	0.540955	9.71E-83
SERTAD2	FBXO11	0.538475	7.40E-82
SERTAD2	VPS54	0.524786	4.06E-77
SERTAD2	TET3	0.518529	5.05E-75
SERTAD2	SOS1	0.510108	2.85E-72
SERTAD2	PPP3R1	0.496853	4.28E-68
SERTAD2	USP34	0.491508	1.84E-66
SERTAD2	SOCS5	0.490345	4.14E-66
SERTAD2	ZFR	0.486352	6.51E-65
SERTAD2	EHBP1	0.481893	1.35E-63
SERTAD2	ACTR2	0.479296	7.77E-63
SERTAD2	ADAM17	0.47055	2.50E-60
SERTAD3	BLVRB	0.574	3.15E-95
SERTAD3	RAB4B	0.550588	3.09E-86
SERTAD3	SERTAD1	0.513417	2.41E-73
SERTAD3	PAFAH1B3	0.512691	4.15E-73
SERTAD3	SMIM22	0.506362	4.51E-71
SERTAD3	COQ8B	0.50366	3.23E-70
SERTAD3	NDUFA2	0.499625	5.93E-69
SERTAD3	TMC4	0.491621	1.70E-66
SERTAD3	MFSD5	0.487272	3.46E-65
SERTAD3	SIGIRR	0.486999	4.18E-65
SERTAD3	SYNE4	0.483666	4.07E-64
SERTAD3	PYM1	0.480988	2.49E-63
SERTAD3	SPINT2	0.475926	7.33E-62
SERTAD4	DIEXF	0.473521	3.58E-61
SERTAD4	SFT2D2	0.459773	2.45E-57
SERTAD4	TIPRL	0.443549	4.95E-53
SERTAD4	DESI2	0.44246	9.44E-53
SERTAD4	TAF5L	0.441538	1.63E-52
SERTAD4	RAP2A	0.418361	8.58E-47

SERTAD4	NFIB	0.402662	3.65E-43
SERTAD4	GNB4	0.400838	9.35E-43
SERTAD4	SPRTN	0.395922	1.15E-41
SERTAD4	AIF1L	0.386955	1.01E-39
SERTAD4	ESRP1	0.386833	1.07E-39
SERTAD4	TMEM65	0.386468	1.28E-39
SERTAD4	SOX4	0.384003	4.25E-39
CDCA4	KIFC1	0.618229	2.41E-114
CDCA4	VRK1	0.610705	7.06E-111
CDCA4	CDC20	0.600876	1.74E-106
CDCA4	CDT1	0.600145	3.63E-106
CDCA4	AURKB	0.576938	2.08E-96
CDCA4	PLK1	0.575009	1.24E-95
CDCA4	MCM7	0.574642	1.74E-95
CDCA4	HJURP	0.574329	2.33E-95
CDCA4	CCNB2	0.570678	6.58E-94
CDCA4	STMN1	0.568997	3.02E-93
CDCA4	PTTG1	0.556712	1.61E-88
CDCA4	MELK	0.555257	5.67E-88
CDCA4	NDC80	0.551082	2.03E-86

Table S3. Top correlated genes and association with TRIP-Brs (Edge) in normal breast tissues

Source	Target	Score	p-value
SERTAD1	UBE2S	0.588035	2.70E-21
SERTAD1	PNO1	0.55182	1.88E-18
SERTAD1	PSMC4	0.542242	9.35E-18
SERTAD1	BCL3	0.531769	5.09E-17
SERTAD1	NXT1	0.530401	6.32E-17
SERTAD1	TSSC4	0.529438	7.36E-17
SERTAD1	TNIP2	0.521942	2.37E-16
SERTAD1	CLTB	0.520549	2.93E-16
SERTAD1	SNRPD1	0.517958	4.36E-16
SERTAD1	TEAD4	0.516684	5.28E-16
SERTAD1	ATG101	0.514237	7.64E-16
SERTAD1	BAG3	0.513574	8.44E-16
SERTAD1	SH2B2	0.51216	1.04E-15
SERTAD2	PPP1R3B	0.719626	1.97E-35
SERTAD2	FEM1B	0.695018	3.31E-32
SERTAD2	AFTPH	0.684811	5.80E-31
SERTAD2	SAMD4B	0.657903	6.46E-28
SERTAD2	ZNF609	0.640244	4.43E-26
SERTAD2	REL	0.634766	1.55E-25
SERTAD2	HSPA4L	0.620789	3.42E-24
SERTAD2	ARHGAP5	0.614358	1.35E-23
SERTAD2	MCPH1	0.61137	2.52E-23
SERTAD2	MED29	0.600308	2.43E-22
SERTAD2	TBC1D22B	0.594262	8.05E-22

SERTAD2	MAP3K14	0.594217	8.13E-22
SERTAD2	DENND4A	0.591072	1.50E-21
SERTAD3	HES1	0.664828	1.14E-28
SERTAD3	DUSP5	0.601201	2.03E-22
SERTAD3	PAK4	0.58694	3.33E-21
SERTAD3	PAF1	0.581964	8.57E-21
SERTAD3	TMEM116	0.578675	1.59E-20
SERTAD3	IRX5	0.578544	1.63E-20
SERTAD3	C16orf74	0.57839	1.67E-20
SERTAD3	ELF3	0.576326	2.45E-20
SERTAD3	BHLHE40	0.57528	2.97E-20
SERTAD3	IRX3	0.568278	1.06E-19
SERTAD3	CFAP53	0.56443	2.11E-19
SERTAD3	ARMCX6	0.563192	2.63E-19
SERTAD3	DYNLRB2	0.560511	4.21E-19
SERTAD4	FAM83F	0.784544	6.92E-46
SERTAD4	ZNF33B	0.765524	1.79E-42
SERTAD4	ARHGEF37	0.7562	6.44E-41
SERTAD4	FAM83H	0.752927	2.18E-40
SERTAD4	SH3BP4	0.746523	2.25E-39
SERTAD4	CAMSAP3	0.742628	8.97E-39
SERTAD4	KIAA1522	0.740451	1.93E-38
SERTAD4	PTK6	0.737636	5.11E-38
SERTAD4	SGSM2	0.730594	5.55E-37
SERTAD4	TJP3	0.729883	7.04E-37
SERTAD4	GGT6	0.724326	4.37E-36
SERTAD4	ZNF185	0.72302	6.66E-36
SERTAD4	MARVELD2	0.720821	1.35E-35
CDCA4	HIST3H2A	0.61971	4.32E-24
CDCA4	TMEM121	0.575521	2.84E-20
CDCA4	THOP1	0.571401	6.04E-20
CDCA4	GATA3	0.570597	6.99E-20
CDCA4	FZR1	0.566767	1.39E-19
CDCA4	SLC44A3	0.56109	3.80E-19
CDCA4	TLE6	0.55596	9.28E-19
CDCA4	LYPD3	0.555795	9.54E-19
CDCA4	PAK4	0.554909	1.11E-18
CDCA4	SPINT2	0.549332	2.87E-18
CDCA4	FAM227B	0.54886	3.11E-18
CDCA4	EPN3	0.548767	3.16E-18
CDCA4	ILDR1	0.543945	7.06E-18

Table S4. GeneSet Analysis: Co-expressed Genes by coexpedia

Rank	Gene Symbol and Name	Score
1	PRC1 (protein regulator of cytokinesis 1)	21.901
2	CDCA4 (cell division cycle associated 4)	9.974
3	BRCA2 (breast cancer 2)	9.306
4	TGFBR2 (transforming growth factor beta receptor II)	9.220
5	EBF1 (early B-cell factor 1)	8.485
6	MDM4 (MDM4, p53 regulator)	7.001
7	RCCD1 (RCC1 domain containing 1)	5.002
8	ITPR1 (inositol 1,4,5-trisphosphate receptor, type 1)	4.992
9	TCF7L2 (transcription factor 7-like 2 (T-cell specific, HMG-box)	4.720
10	ERBB4 (erb-b2 receptor tyrosine kinase 4)	4.419
11	AP4B1 (adaptor related protein complex 4, beta 1 subunit)	4.009
12	DCLRE1B (DNA cross-link repair 1B)	4.009
13	NEK10 (NIMA-related kinase 10)	3.902
14	TOX3 (TOX high mobility group box family member 3)	3.758
15	MLLT10 (myeloid/lymphoid/mixed-lineage leukemia; translocated to, 10)	3.556
16	ZMIZ1 (zinc finger, MIZ-type containing 1)	3.225
17	CCDC170 (coiled-coil domain containing 170)	2.919
18	FGFR2 (fibroblast growth factor receptor 2)	2.542
19	PKNOX2 (PBX/knotted 1 homeobox 2)	2.356
20	CHST9 (carbohydrate (N-acetylgalactosamine 4-0) sulfotransferase 9)	2.338
21	SERTAD1 (SERTA domain containing 1)	2.131
22	ELL (elongation factor RNA polymerase II)	2.131
23	MAP3K1 (mitogen-activated protein kinase kinase kinase 1, E3 ubiquitin protein ligase)	2.126
24	SERTAD2 (SERTA domain containing 2)	1.912
25	CCDC88C (coiled-coil domain containing 88C)	1.695
26	PDE4D (phosphodiesterase 4D)	1.574
27	SLC4A7 (solute carrier family 4, sodium bicarbonate cotransporter, member 7)	1.564
28	ZC3H11A (zinc finger CCCH-type containing 11A)	1.319
29	FTO (fat mass and obesity associated)	1.099

Table S5. GeneSet Analysis: Gene Ontology - Biological Process

*** Enriched terms by p-value < 0.05 by coexpedia**

Rank	GO Acc. and Desc.	p-value
1	GO:0001568 (blood vessel development)	4.801e-5
2	GO:0008284 (positive regulation of cell proliferation)	5.186e-5
3	GO:0048625 (myoblast fate commitment)	1.542e-3
3	GO:0090335 (regulation of brown fat cell differentiation)	1.542e-3
3	GO:1990426 (homologous recombination-dependent replication fork processing)	1.542e-3
3	GO:2000366 (positive regulation of STAT protein import into nucleus)	1.542e-3
3	GO:0032350 (regulation of hormone metabolic process)	1.542e-3
3	GO:0033688 (regulation of osteoblast proliferation)	1.542e-3
3	GO:0031098 (stress-activated protein kinase signaling cascade)	1.542e-3
3	GO:0010883 (regulation of lipid storage)	1.542e-3
11	GO:0000910 (cytokinesis)	1.643e-3
12	GO:0018108 (peptidyl-tyrosine phosphorylation)	1.728e-3
13	GO:0042245 (RNA repair)	3.082e-3
13	GO:0045023 (G0 to G1 transition)	3.082e-3
13	GO:0044334 (canonical Wnt signaling pathway involved in positive regulation of epithelial to mesenchymal transition)	3.082e-3
13	GO:0050849 (negative regulation of calcium-mediated signaling)	3.082e-3
13	GO:0000022 (mitotic spindle elongation)	3.082e-3
13	GO:0032754 (positive regulation of interleukin-5 production)	3.082e-3
13	GO:0035552 (oxidative single-stranded DNA demethylation)	3.082e-3
20	GO:2000675 (negative regulation of type B pancreatic cell apoptotic process)	4.620e-3
20	GO:0035553 (oxidative single-stranded RNA demethylation)	4.620e-3

Table S6. GeneSet Analysis: Disease Ontology

*** Enriched terms by p-value < 0.05**

Rank	MDO ID and Name	p-value
1	DOID:4241 (malignant neoplasm of breast)	1.178e-5
2	DOID:5683 (hereditary breast ovarian cancer)	3.567e-4
3	DOID:2985 (chronic rejection of renal transplant)	6.025e-4
4	DOID:768 (retinoblastoma)	7.074e-4
5	DOID:3114 (serous cystadenocarcinoma)	1.401e-3
6	DOID:9370 (exophthalmos)	1.542e-3
6	DOID:6586 (juvenile breast carcinoma)	1.542e-3
6	DOID:14705 (Pfeiffer syndrome)	1.542e-3
6	DOID:2339 (Crouzon syndrome)	1.542e-3
6	DOID:2531 (hematologic cancer)	1.542e-3
11	DOID:8719 (in situ carcinoma)	1.748e-3
12	DOID:10603 (glucose intolerance)	2.088e-3
13	DOID:162 (cancer)	2.588e-3
14	DOID:2144 (malignant neoplasm of ovary)	2.904e-3
15	DOID:4552 (large cell carcinoma)	3.082e-3
15	DOID:1961 (fallopian tube cancer)	3.082e-3
15	DOID:10688 (hypertrophy of breast)	3.082e-3
15	DOID:5583 (giant cell carcinoma)	3.082e-3
15	DOID:5744 (serous adenocarcinoma of the ovary)	3.082e-3
15	DOID:172 (clear cell acanthoma)	3.082e-3
15	DOID:174 (acanthoma)	3.082e-3
15	DOID:14768 (Saethre-Chotzen syndrome)	3.082e-3
23	DOID:1240 (leukemia)	3.793e-3
24	DOID:1749 (squamous cell carcinoma)	4.394e-3
25	DOID:0050466 (Loeys-Dietz syndrome)	4.620e-3
25	DOID:1963 (fallopian tube carcinoma)	4.620e-3
27	DOID:3007 (ductal carcinoma)	7.405e-3
28	DOID:6741 (bilateral breast cancer)	7.689e-3
28	DOID:14323 (marfan syndrome)	7.689e-3
28	DOID:11717 (neonatal diabetes mellitus)	7.689e-3
31	DOID:3459 (breast carcinoma)	8.972e-3

Table S7. TRIP-Brs and visualize the correlation of its expression with immune infiltration level in patients with BRCA. Correlation between *TRIP-Brs* in BRCA expression and abundance of immune infiltrates was statistically significant. The table is showing the purity-corrected partial Spearman's correlation and statistical significance. (TIMER Database).

Clinicopathologic variable	Correlation	*p*-Value	Significant
**SERTAD1 or TRIP-Br1**			
Purity	-0.176	2.34e-08	
B Cell	-0.11	5.83e-04	
CD8+ T Cell	-0.128	5.79e-05	
CD4+ T Cell	0.076	1.85e-02	
Macrophage	-0.027	3.92e-01	
Neutrophil	-0.001	9.65e-01	
Dendritic Cell	0.02	5.31e-01	
**SERTAD2 or TRIP-Br2**			
Purity	-0.057	7.43e-02	
B Cell	0.095	2.93e-03	
CD8+ T Cell	0.32	1.17e-24	
CD4+ T Cell	0.211	4.04e-11	
Macrophage	0.205	8.12e-11	
Neutrophil	0.286	2.40e-19	
Dendritic Cell	0.211	5.14e-11	
**CDCA4 or TRIP-Br3**			
Purity	0.128	5.29e-05	
B Cell	0.061	5.56e-02	
CD8+ T Cell	-0.029	3.58e-01	
CD4+ T Cell	0.09	5.27e-03	
Macrophage	0.154	1.28e-06	
Neutrophil	0.063	5.23e-02	
Dendritic Cell	0.076	1.96e-02	
**SERTAD3 or TRIP-Br4**			
Purity	0.121	1.26e-04	
B Cell	-0.126	8.31e-05	
CD8+ T Cell	-0.065	4.36e-02	
CD4+ T Cell	-0.01	7.62e-01	
Macrophage	-0.025	4.43e-01	
Neutrophil	-0.082	1.12e-02	
Dendritic Cell	-0.118	2.54e-04	
**SERTAD4 or TRIP-Br5**			
Purity	0.054	8.78e-02	
B Cell	0.076	1.71e-02	
CD8+ T Cell	0.238	5.34e-14	
CD4+ T Cell	0.105	1.16e-03	
Macrophage	0.208	4.30e-11	
Neutrophil	0.173	7.99e-08	
Dendritic Cell	0.142	1.10e-05	
